# Two-Component System Genes in *Brassica napus*: Identification, Analysis, and Expression Patterns in Response to Abiotic and Biotic Stresses

**DOI:** 10.3390/ijms242417308

**Published:** 2023-12-09

**Authors:** Hongfang Liu, Nian Liu, Chen Peng, Jiaquan Huang, Wei Hua, Zhengwei Fu, Jing Liu

**Affiliations:** 1 Key Laboratory of Biology and Genetic Improvement of Oil Crops, Oil Crops Research Institute of the Chinese Academy of Agricultural Sciences, Ministry of Agriculture and Rural Affairs, Wuhan 430062, China; liuhongfang01@caas.cn (H.L.);; 2 School of Breeding and Multiplication, Sanya Institute of Breeding and Multiplication, Hainan University, Sanya 570208, China; 3 Hubei Hongshan Laboratory, Wuhan 430070, China

**Keywords:** rapeseed, two-component system, phylogeny, genetic diversity, expression

## Abstract

The two-component system (TCS), consisting of histidine kinases (HKs), histidine phosphotransfer proteins (HPs) and response regulators (RRs) in eukaryotes, plays pivotal roles in regulating plant growth, development, and responses to environment stimuli. However, the TCS genes were poorly characterized in rapeseed, which is an important tetraploid crop in Brassicaceae. In this work, a total of 182 BnaTCS genes were identified, including 43 HKs, 16 HPs, and 123 RRs, which was more than that in other crops due to segmental duplications during the process of polyploidization. It was significantly different in genetic diversity between the three subfamilies, and some members showed substantial genetic differentiation among the three rapeseed ecotypes. Several hormone- and stress-responsive cis-elements were identified in the putative promoter regions of BnaTCS genes. Furthermore, the expression of BnaTCS genes under abiotic stresses, exogenous phytohormone, and biotic stresses was analyzed, and numerous candidate stress-responsive genes were screened out. Meanwhile, using a natural population with 505 *B. napus* accessions, we explored the genetic effects of BnaTCS genes on salt tolerance by association mapping analysis and detected some significant association SNPs/genes. The result will help to further understand the functions of TCS genes in the developmental and stress tolerance improvement in *B. napus.*

## 1. Introduction

In both prokaryotes and eukaryotes, protein phosphorylation is the key mechanism regulating signal transduction pathways, which is crucial in adverse environmental stimulus responses. By inducing a conformational change in the regulatory domain that results in the activation of an associated domain, phosphorylation may affect the stimulus response. Many signal transduction pathways employ a so-called “two-component system (TCS)” via phosphorylation between histidine (His) and aspartic acid (Asp) residues [1,2]. TCS was originally identified in *Escherichia coli* [3], and since then has been widely reported in prokaryotes [4]. In bacteria, the TCS is composed of a membrane-associated histidine protein kinase (HK) and a cytoplasmic response regulator (RR), following simple His-to-Asp auto-phosphorelay [1]. In response to changes in environmental conditions, HK proteins primarily sense stress signals and autophosphorylate the His residue in the HK domain. Afterward, the phosphate group is transferred to a conserved Asp residue within the receiver (Rec) domain of RR proteins. Many RRs are transcription factors and those with phosphorylation can convert external stimuli into internal signals by mediating downstream signaling. In eukaryotes, the TCS has evolved a more complex multi-step phosphorylation system that employs a hybrid HK with both histidine kinase and receiver domains, a histidine-containing phosphotransfer protein (HP), and an RR. The corresponding signaling system transfers phosphates in the sequence “His-Asp-His-Asp” [2]. The HP protein acts as a linker between HK and RR [5]. It is responsible for phosphate transfer between the hybrid HK and RR and enables the four-step phosphotransfer to take place. The multi-step TCS in eukaryotes provides multiple regulatory checkpoints for signal cross-talk and a greater number of potential steps for regulation, which contribute to improving the complexity and accuracy of the regulation [4].

Based on the sequence signature, functional characteristics, and prevalence among species in evolution, TCS genes could be broadly classified into several subgroups in plants. HKs are comprised of three subfamilies (cytokinin receptor, ethylene receptor, and phytochrome) and three additional ungrouped genes (AHK1-like, CKI1-like, and CKI2/AHK5-like). Several conserved residues in the transmitter domain, including the autophosphorylation site (a His residue), are crucial for HK activity [4]. Therefore, the phytochromes and three ethylene receptors (ETR2-, ERS2-, and EIN4-like genes) are referred to as divergent HKs due to the lack of these motifs. In addition, the cytokinin receptors involve a conserved cyclase/histidine kinase-associated sensory extracellular (CHASE) domain, which is the putative binding site for cytokinin molecules [6], ethylene receptors share an ethylene binding domain [7], and phytochromes contain photosensory core domains (PAS-GAF-PHY) at the N-terminal [8]. HPs contain both HP and pseudo-HP. A highly conserved XHQXKGSSXS motif in HPs is necessary for the transfer of the phosphate group from the Rec domain of HKs to the Rec domain of RRs [5]. However, the His residue is missing in AHP6/PHP1-like genes, resulting in them being called pseudo-His-containing phosphotransfer proteins (pseudo-HP). RRs involve four subgroups (type-A, type-B, type-C, and pseudo-RRs), and all contain a phospho-accepting Rec domain [4]. Type-A RRs are cytokinin response proteins with short N- and C-terminal extensions [9]. Type-B RRs are transcription factors that contain long C-terminal extensions with a Myb-like DNA binding domain [10]. Type-C RRs have domain structures similar to type-A RRs but lack long C-terminal extensions. Another divergent class of RRs misses a D residue, which is necessary for phosphorylation, so they are known as pseudo-RRs (PRRs). To date, TCS genes have been whole-genome identified in many plants, including *Arabidopsis* [2,5,11], rice [12], soybean [13], maize [14], Chinese cabbage [15], wheat [16], sorghum [17], sweet potato [18], and cucumber [19]. Most TCS members are conservative except for a few species missing the type-C RR genes.

The TCS-mediated signaling pathway plays a significant role in regulating plant growth and development [11]. It has been extensively studied that TCS is related to cytokinin (CK) signaling transduction which participates in numerous aspects of plant lifecycle [6,20,21,22,23,24]. Analysis of loss-of-function mutants has revealed that three cytokinin receptors (AHK2, AHK3, and AHK4), four AHPs (AHP1, AHP2, AHP3, and AHP5), and type-B ARRs act as positive regulators in cytokinin signal transduction [2]. In the *Arabidopsis* triple mutant *hk2 hk3 hk4* and rice double mutant *hk5 hk6*, they exhibited reduced sensitivity to exogenous cytokinin, and the development of root, shoot, leaf, and inflorescence meristem was markedly inhibited [22,24]. Similarly, the *Arabidopsis* triple mutant *arr1 arr10 arr12* formed narrower inflorescence stems and shorter siliques, and the expression of most cytokinin-responsive genes was regulated in the mutant [25]. These reports indicate that TCS such as HKs and RRs regulate plant growth and development by mediating cytokinin signaling. TCS is also demonstrated to be closely implicated in response to biotic and abiotic stresses [26,27,28,29,30,31,32,33,34,35]. *Arabidopsis* HK1 (AtHK1) was known to be a positive regulator of osmotic stress responses and abscisic acid (ABA) signaling. The germination rates of *athk1* mutants were higher in the presence of ABA, indicating that *ahk1* mutants were ABA-insensitive. Moreover, under drought/salt stress, fewer *ahk1* plants survived than wild-type plants, suggesting the drought/salinity sensitivity of the *ahk1* mutant [26]. In contrast, three cytokinin receptor HKs (AtHK2-4) were recognized as negative regulators in ABA, salt, and drought signaling pathways based on the phenotypic analysis of the single, double, and triple mutants [26,27]. AtHK2 and AtHK3 also play a role in cold stress response [28]. In addition, AtHK5 regulates both salt stress tolerance and resistance to pathogens. The loss of *AtHK5* function increases susceptibility to the biotrophic bacterium *Pst*DC3000 and the necrotrophic fungus *B. cinerea* [29]. Most AtHPs have been proved to be redundant positive regulators of cytokinin signaling [30,31], and they (AtHP2-5) negatively regulate drought stress response which is similar to cytokinin receptors [32,33]. Moreover, AtHP2, AtHP3, and AtHP5 are redundantly involved in mediating the cold signaling as the regulated downstream of AtHK2 and AtHK3 [30]. Three type-B RRs, AtRR1, AtRR10, and AtRR12, negatively regulate plant responses to drought [34]. Three pseudo-response regulators, AtPRR5, AtPRR7, and AtPRR9, are negative regulators of drought, salinity, and cold [35]. Similarly, TCS genes have also been demonstrated to regulate environmental stresses in several other plants such as rice and cotton [36,37,38,39].

Rapeseed (*Brassica napus*) is one of the most important oilseed crops in Brassicaceae and is also utilized as a source of protein feed and industrial raw materials. It is an allotetraploid crop with two diploid parents, *Brassica rapa* and *Brassica oleracea,* both of which have undergone a whole-genome triplication (WGT) event, and thus, rapeseed serves as an ideal polyploid model for studying gene family expansion as well as gene sequence and function divergence. The complex genome indicates that rapeseed may have evolved a more complex TCS system. However, TCS genes have not been systematically investigated in rapeseed. In this study, genome-wide identification and characterization of TCS genes in rapeseed were conducted, including sequence signature, gene structure, functional domain, classification, and phylogenetic relationship analysis. We further performed an analysis of TCS gene expression profiles in various organs and investigated the response patterns to adverse environmental stresses. Several organ-specific expression genes and numerous candidate stress-responsive genes were screened out. In addition, genetic polymorphism analysis and family-based association analysis with salt tolerance coefficient of BnaTCS genes were conducted based on population sequencing data. Our study provides insight into TCS in a polyploid plant on a genome-wide scale for the first time and provides a foundation for further elucidating the roles of TCS genes in plant growth and stress regulatory networks.

## 2. Results

### 2.1. Identification of Two-Component System Genes in Brassica napus and Its Two Diploid Progenitors

A total of 532 putative BnaTCS genes were identified based on blastp [40] searching using TCS protein sequences from five plant species (*Arabidopsis*, Chinese cabbage, soybean, sorghum, and rice) as queries. Subsequently, these putative sequences were filtered using the hmmsearch program of HMMER 3.0 [41], and further confirmed by checking the presence of the conserved structural characteristics and specific domains of TCS elements using different domain databases including Pfam [42], CDD [43] and SMART [44]. After the removal of all the partial and redundant sequences, 43 HK, 16 HP, and 123 RR genes were identified in *B. napus* (Table S1). To determine the evolutionary origin of BnaTCS genes in a follow-up analysis, we also identified TCS genes in its two diploid progenitors. Finally, 22 HK, 8 HP, and 65 RR genes were identified in *Brassica oleracea* (Table S2), and 21 HK, 8 HP, and 59 RR genes were identified in *Brassica rapa* (Table S3), which were three more than identified in a previous study [15] because of the advancement of genome assembly (Table S4). TCS proteins identified in the three Brassica species were named based on the homologous genes in *Arabidopsis*. Letters were added in order at the end of the name when more than one TCS gene corresponded to one *Arabidopsis* TCS gene.

#### 2.1.1. Histidine Kinase Proteins in Rapeseed

**Figure 1:**
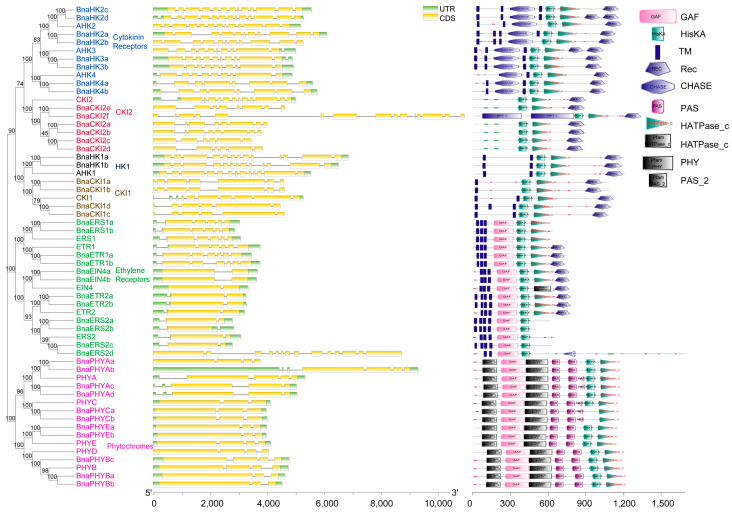
Phylogenetic relationships, gene structure, and protein domains of HK(L)s of *B. napus* and *Arabidopsis*. HK(L)s in *Arabidopsis* are marked with solid circles in front of the gene name and different subgroups of HK(L)s are highlighted with different colors and labeled alongside. In gene structure, the UTRs and CDS are indicated by green and yellow boxes, respectively. In protein domains annotated and displayed by SMART, different colors and shapes represent different domains.

In total, 43 HKs (BnaHKs/BnaHKLs) were found in *Brassica napus*, which could be categorized into 6 subgroups: 12 ethylene receptors, 11 phytochrome photoreceptors, 8 cytokinin receptors, 2 AHK1-like, 4 CKI1-like and 6 CKI2/AHK5-like genes (Figure 1, Table S1). Among these proteins, 24 BnaHKs possessed a typical conserved HK domain that contained five signature motifs, namely H, N, G1, F, and G2 (Figure S1A). The key feature is the conserved His in the H motif, and the other four define the nucleotide-binding cleft [5]. Correspondingly, the remaining 19, consisting of 8 ethylene receptors (4 BnaERS2s, 2 BnaETR2s, and 2 BnaEIN4s) and 11 phytochrome photoreceptors, were identified as histidine protein kinase-like genes (HKLs) as their His-kinase transmitter (HK) domain was divergent. Domain analysis confirmed that almost all BnaHK(L)s (except for BnaPHYCa and BnaPHYCb) contained a HisKa domain but most BnaHKLs missed the conserved His phosphorylation site through multiple-sequence alignment. Meanwhile, the other four incomplete or missing motifs suggested a loss of function of histidyl-aspartyl phosphorelays.

In rapeseed, there were at least two copies (except PHYD) of HK(L)s and they contained the same domains as homologs of *A. thaliana*. The cytokinin receptor family was composed of 8 histidine kinases: 4 BnaHK2s, 2 BnaHK3s, and 2 BnaHK4s. They all comprised 2–3 trans-membrane (TM) domains surrounding cyclases/histidine kinases associated sensing extracellular (CHASE) domain followed by HisKa domain, HATPase_c, and a response regulator Rec domain (Figure 1). Of these, the CHASE and TM domains were demonstrated to be specific for membrane-associated cytokinin recognition and binding [4]. The subcellular localization prediction showed that all eight proteins mainly localized in the plasma membrane (PM), indicating that TM domains were functional in BnaHKs. AHK1-like, CKI1-like, and CKI2-like proteins had domain structures similar to cytokinin receptors, but lacked the CHASE domain. AHK1 and CKI1 homologs were also located in PM. However, CKI2 homologs were predicted to be mainly located in the nucleus and cytoplasmic due to the lack of the TM domain. The ethylene receptor family of rapeseed was involved in two subfamilies with four HK genes (two BnaERS1 and two BnaETR1) in subfamily 1, and eight HKL genes (two BnaERS2, four BnaETR2, and two BnaEIN4) in subfamily 2. They all contain 3–4 TMs, which comprise the ethylene-binding domain, followed by a cyclic GMP adenylyl cyclase FhlA (GAF), and HK domain. Besides, both BnaETR1s, BnaETR2s, and BnaEIN4s possessed additional HATPase_c and REC domains at the C-terminal. Similar to *B. rapa*, an ERS2-like gene BnaERS2d (BrHKL4 in *B. rapa*) was presumed to be formed by the fusion of two genes and contained an additional MATH domain [15]. Evidence showed that the HK activity might not alter ethylene signal transduction [45], but could trigger the TCS and promote plant growth in *Arabidopsis* [46]. Phytochromes are photoreceptors that allow plants to respond to light stimuli [47]. A total of 11 BnaPHYs, which belonged to 5 PHY subfamilies, were identified in rapeseed. They all contained PHY (chromophore-binding), GAF, and PAS (signal sensor) domains, forming a PAS-GAF-PHY tri-domain that acted on absorbing light and inducing conformational changes [47]. Unlike other HKs, all the BnaPHYs lacked TM and Rec domains and contained a divergent HK domain. As a result, these 11 proteins could not be involved in HK phosphorylation and were predicted to be mainly located in the chloroplast, cytoplasm, and nucleus (Table S1).

#### 2.1.2. Histidine Phosphotransfer Proteins in Rapeseed

HP proteins are the signal mediators in multi-step His-Asp-His-Asp phosphorelay which transfers the phosphoryl group from HKs to RRs [2]. We identified 16 HP proteins in rapeseed that could be divided into 2 subgroups: 14 authentic HPs and 2 pseudo-HP proteins based on the presence of His phosphorylation site (Table S1). All Bna(P)HPs exhibited high protein sequence similarities to *Arabidopsis* homologs with an identity ranging from 88% to 95%, and they only had an Hpt or pseudo-Hpt domain (Figure S2). Compared to BnaPHP1s, the BnaHPs encoded a conserved motif XHQXKGSSXS with a His phosphorylation site (Figure S1B). No TM domain was identified in BnaHPs, and subcellular localization prediction showed that the 16 Bna(P)HP proteins were mainly located in the chloroplast, mitochondrion, or nucleus.

#### 2.1.3. Response Regulators in Rapeseed

RR is the terminal component of TCS that functions as a signal executor to regulate the final responses to environmental stimuli. On the basis of the conserved domains, phylogenetic and functional analyses, the RR family members could be divided into four subfamilies: type-A RR, type-B RR, type-C RR, and PRR. They all contain a phospho-accepting receiver (Rec) domain, which has been found with two conserved Asp and lysine (K) residues in *Arabidopsis.*

In this study, 123 RRs, including 43 type-A RRs, 38 type-B RRs, 10 type-C RRs, and 32 pseudo-RRs (PRRs), were identified in *B. napus* (Table S1). They were homologs of 31 *A. thaliana* (P)RRs (excluding ARR13 and PRR8). The type-A RRs had relatively short ORF lengths (less than 300 amino acids) with 4 or 5 exons in the coding region, and showed more than 72% similarities to their homologs in *Arabidopsis*. Most type-A BnaRRs were predicted to be located in the nucleus followed by cytoplasm, whereas six BnaRR5 proteins showed subcellular localization in peroxisome and two BnaRR15 proteins in Golgi. All type-A BnaRRs were found to have conserved Rec domain including three invariant amino acid residues (D-D-K) except for BnaRR4c and BnaRR4d, which lack the central aspartate site (D) and the C-terminal lysine (K) (Figure S3; Table S1). These three residues were all important for their phospho-accepting function. For the 38 type-B RRs, 33 proteins had long C-terminal extension with a Myb-like domain, while the other 5 (BnaRR1f, BnaRR1g, BnaRR10c, BnaRR12c, and BnaRR23) missed the Myb-like domain, suggesting that they had lost their function as transcription factors (Figure S4; Table S1). In addition, all type-B RR proteins except BnaRR12c and BnaRR1c retained these three conserved residues. Almost all of the 33 type-B BnaRRs with a complete structure were predicted to be localized in the nucleus, except that BnaRR11a and BnaRR11b were located in the chloroplast. In rapeseed, type-C RRs were the rarest members of the RR family. However, they possessed a relatively large number of copies. For example, ARR24 had six homologous genes, and ARR22 had four. They possessed only the Rec domain and short N-terminus. Subcellular localization prediction showed that the type-C RR proteins were mainly located in cytoplasmic. PRRs, also known as divergent RRs, could be divided into two subgroups: Clock PRR and type-B PRR. There were 19 Clock PRRs and 13 type-B PRRs in *B. napus.* They also contained a Rec domain, but it was atypical because the N-terminal conserved D site was substituted with several other amino acid residues and the central D site was replaced by glutamate residue (E), except BnaPRR6s by asparagine (N). In addition, type-B BnaRRs were featured with a Myb-like domain (except BnaPRR6), and Clock BnaPRRs were featured with a CCT domain. Almost all BnaPRRs were predicted to localize in the nucleus (Table S1). Notably, BnaPRR9d contained an additional cation_efflux domain, which belonged to the zinc transporter (ZAT) and cation diffusion facilitator (CDF) families (Figure S4). Members of this family are integral membrane proteins and are found to increase tolerance to divalent metal ions such as cadmium, zinc, and cobalt [48]. Notably, BnaPRR9b had seven predicted transmembrane domains and was predicted to be located on the plasma membrane. Based on sequence alignment and gene structure analyses, we proposed that *BnaPRR9b* was formed by the fusion of two genes (Figure S5).

### 2.2. Phylogenetic Analysis

**Figure 2:**
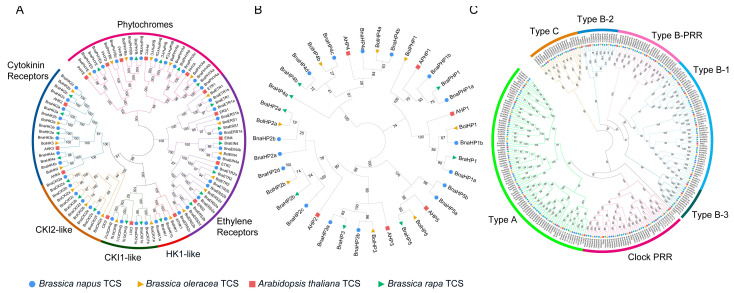
Phylogenetic relationship of TCS genes in *B. napus*, *B rapa*, *B. oleracea*, and *Arabidopsis*. (**A**) Phylogenetic tree of histidine kinases (HKs). (**B**) Phylogenetic tree of histidine phosphotransfer proteins (HPs). (**C**) Phylogenetic tree of response regulators (RRs). The genes from different species are marked with solid circle, trangle, and square in different colors in front of gene name and different subgroups are highlighted by different colors and labeled alongside. The numbers aside the branches are associated bootstrap values.

To investigate the phylogenetic relationship of TCS genes between *B. napus, Arabidopsis*, and diploid progenitors of rapeseed, a neighbor-joining (NJ) tree was constructed for HK, HP, and RR subfamily, respectively, by using the protein sequences of the identified TCS genes. According to the topology of the trees (Figure 2), the orthologs among four genomes and the paralogs in one species were closely related, and each TCS gene in *Arabidopsis* had at least one homologous gene in three Brassica species except PHYD, ARR13, and PRR8, indicating that HK genes were evolutionarily conserved. TCS genes in rapeseed were all clustered closely with *Arabidopsis* TCS genes, implying that they have similar functions to *A. thaliana* counterparts. Furthermore, in nearly all subgroups, there was a one-to-one correspondence between TCS genes from subgenome A of *B. napus* and *B. rapa* (BnaA-Bra)*,* as well as TCS genes from subgenome C of *B. napus* and *B. oleracea* (BnaC-Bol). Only the TOC1 subgroup was asymmetric because there were two TOC1 homologous genes in *B. rapa* but only *BnaTOC1a* was located on subgenome A_n_ (A03), probably due to gene loss during speciation.

HKs were divided into six subgroups (cytokinin receptors, CKI2, CKI1, AHK1, ethylene receptors, and phytochromes), in which the cytokinin receptors, CKI2, CKI1, and AHK1 were clustered together in one clade while ethylene receptors and phytochromes were in another two independent clades (Figure 2A). This is consistent with the classification by functional domains they contained and as well as with previous studies [11,18]. Phylogenetic analysis of the HP members showed three clades and six divisions, corresponding to six *Arabidopsis* HP members (Figure 2B). The AHP4 occupied a single clade. The AHP2, AHP3, and AHP5 subgroups were grouped into the same clade, indicating a close relationship between these subgroups (Figure 2A). The pseudo-HP proteins adjoined the AHP1 subgroup and formed another clade with it (Figure 2B). All the 180 RR proteins from four species were grouped into type-A, type-B, type-C, and PRR (Figure 2C), among which type-B RRs could be further divided into four subgroups, namely B-1, B-2, B-3, and B-PRR. All the members of each subgroup were clustered together and formed a clade. Notably, each subfamily contained representative RR proteins from *B. napus*, while several other plants (rice, soybean, maize, tomato, and sweet potato) had no homologs in the type-B-2 subgroup [18]. This suggests that the type-B-2 subgroup may only exist in Cruciferae. Type-A and type-C RRs were clustered into different branches and their genetic distance was not closely related although they shared a similar structure. Pseudo-RR contained two subgroups: type-B-PRR proteins and Clock PRR proteins. Type-B-PRR proteins were clustered in the type-B clade, while Clock PRR proteins had relatively closer phylogenetic relationships with type-A RR members (Figure 2C).

### 2.3. Genomic Distribution, Gene Duplication, and Synteny Analysis

Based on gene position annotation, 182 identified *B. napus* TCS genes were unevenly distributed on each chromosome. In detail, HK(L)s and RRs were mapped on all 19 chromosomes, while HPs were only located on 16 chromosomes. Chromosomes A03 and C03 contained the most members (both with 13 TCS genes), while A08 had only four TCS genes (Table S1).

**Figure 3:**
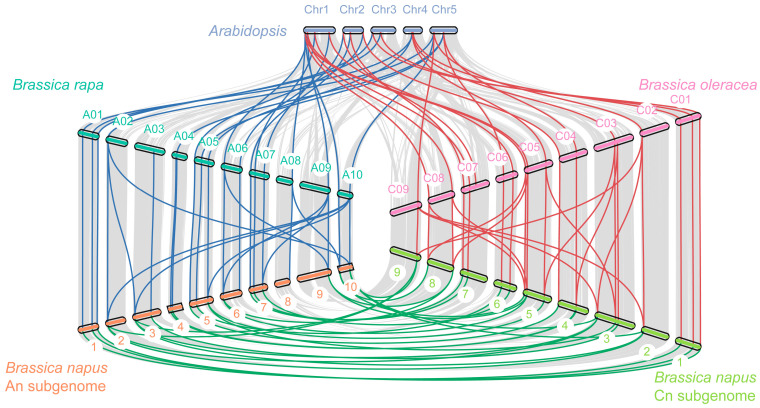
Collinear correlation of TCS genes in *A. thaliana*, *B. rapa*, *B. oleracea*, and *B. napus*. The chromosome names of *A. thaliana*, *B. rapa*, *B. oleracea*, *B. napus* subgenome A, and *B. napus* subgenome C are labeled alongside the chromosome and marked with five colors, respectively, and the corresponding species name is labeled on both sides. The grey lines represent the collinear block within the four genomes and the green, red, and blue lines represent the paralogous/orthologous gene pairs within the TCS members.

Among the plants studied, the most TCS genes were found in *B. napus*. In order to explore the evolutionary history and gene family expansion process of *B. napus* TCS genes, we traced the orthologous and the paralogous gene pairs between and within four species (*A. thaliana*, *B. napus* and its two diploid progenitors: *B. rapa* and *B. oleracea*) (Table 1). Previous studies have revealed that Brassicaceae genomes underwent three whole-genome duplications (named γ, β, and α) and another triplication event after speciation from *A. thaliana* [49], which may result in triplicated orthologous copies in *B. rapa* and *B. oleracea.* However, in this study, there were 90 orthologous gene pairs between *A. thaliana* and *B. rapa* and 91 between *A. thaliana* and *B. oleracea*, which involved 46–77 and 46–80 genes, respectively (Table S5). These data indicated that most TCS genes in *A. thaliana* were inherited as only one or two copies in *B. rapa* and *B. oleracea*, suggesting that a substantial gene loss occurred during the process of polyploidization. Notably, the two BolPHYBs and BraPHYB were more clustered together with PHYB in the phylogenetic tree, but all were detected to be syntenic with *PHYD*. Meanwhile, as the hybrid product, *B. napus* might possess a number of TCS genes that are close to the sum of those in *B. rapa* and *B. oleracea*. As a matter of fact, a total of 419 (202 between Bra-BnaA, and 217 between Bol-BnaC) orthologous gene pairs between rapeseed and its two progenitor species were observed (Figure 3), involving 174 BnaTCS genes (95.6%), which is consistent and expected and verified that majority of the BnaTCS genes were inherited from their progenitors.

We also investigated the gene duplication events in *B. napus* TCS genes. In total, 84 A-A, 88 C-C, and 219 A-C duplication pairs were found, involving a total of 95% of the BnaTCS genes (Table S6). Without regard to A-C pairs, 86% of BnaHK(L)s, 100% of BnaHPs, and 94% of BnaRRs underwent duplication events within subgenome A/C. Moreover, 70.9% of BnaTCS genes (129/182) experienced WGD/segmental duplication, 7.7% of BnaTCS genes (14/182) resulted from transposed duplication, 12.6% of BnaTCS genes (23/182) originated from dispersed duplication, and only two tandem duplicated gene pairs (*BnaPRR6a—BnaPRR6c*, *BnaPRR6e*—*BnaPRR6d*) were present.

To estimate the selection pressure of TCSs, the non-synonymous to synonymous substitution ratios (Ka/Ks) of TCS orthologous genes between *A. thaliana*, *B. rapa*, *B. oleracea*, and *B. napus* were calculated (Table S5). The Ks values ranged from 0.00110683 to 4.90995. Except for 11 orthologous gene pairs (involving 22 genes), the Ka/Ks ratios of all pairs were less than 1, indicating that most of the TCS genes of these 4 species underwent purification selection. In addition, the Ka/Ks ratio of the orthologous gene pairs of HKs between *B. napus* and *B. oleracea* was significantly higher than that between *B. napus* and *B. rapa*, showing that HKs in the C_n_ subgenome had experienced relatively weaker selection pressure than A_n_ during evolution (Table S7, Figure S6).

### 2.4. Natural Variations of the BnaTCS Genes

**Figure 4:**
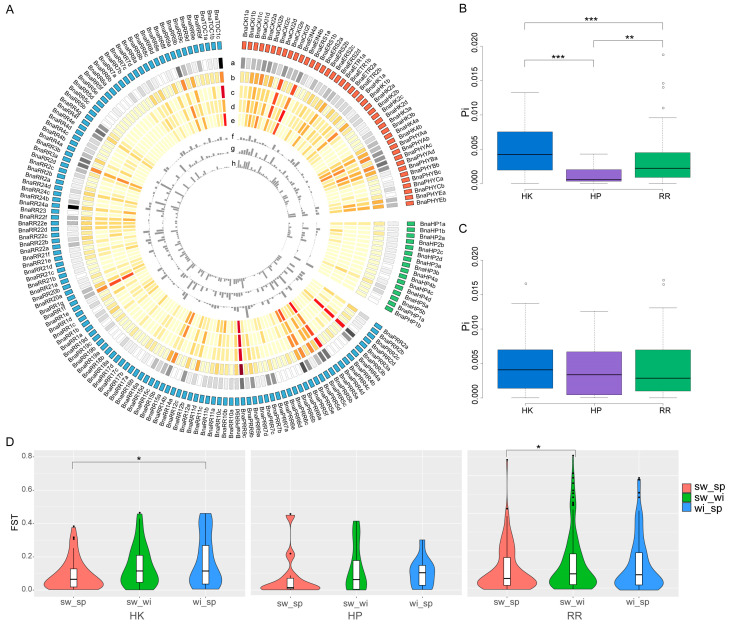
Genetic diversity (π) and fixation index (Fst) between the three ecotypes of each BnaTCS gene. (**A**) Circos plot showing genetic diversity and fixation index of each BnaTCS gene. Circos from outer to inner displayed: a. Heatmap of SNP/small indel density; b. Heatmap of π in whole population; c. Heatmap of π in spring-type accessions; d. Heatmap of π in semi-winter-type accessions; e. Heatmap of π in winter-type accessions; f. Histogram of Fst in semi-winter versus spring ecotypes; g. Histogram of Fst in semi-winter versus winter ecotypes; h. Histogram of Fst in winter versus spring ecotypes. (**B**) Genetic diversity of CDS among three subfamilies members. (**C**) Genetic diversity of the putative promoter regions among three subfamilies members. (**D**) Violin plot of Fst values of three subfamilies. Significant difference are marked with: ‘***’ (*p* < 0.001), ‘**’ (*p* < 0.01), and ‘*’(*p* < 0.05).

Critical sequence polymorphism across the gene and its flanking regions may reflect the evolutionary process of adapting to different environments. To investigate the genetic variations of BnaTCS genes, SNPs and small indels were identified using re-sequencing datasets that contained 525 *B. napus* accessions (including 349 semi-winter types, 77 spring types, and 95 winter types). A total of 7937 and 6535 polymorphism sites were detected in CDS and putative promoter regions of BnaTCS genes, respectively (Table S8), with an average of 28.49 and 35.91 SNPs/small indels per kilobase (kb). Genetic diversity (π) was measured and compared in whole and sub-populations (Figure 4A; Table S9). For the whole group, it ranged from 6.64205 × 10^−6^ (*BnaRR6b*) to 0.0184347 (*BnaPRR9c*), and there was a degree of positive correlation among the density and π value with a correlation coefficient of 0.69 (Figure 4A). The genetic diversity of CDS was significantly different between the three subfamilies (Student’s *t*-test, *p* < 0.01), while BnaHKs had the highest average π value and BnaHPs had the lowest one (Figure 4B). On the contrary, the genetic diversity was similar at putative promoter regions of BnaTCS genes (Figure 4C).

The fixation index (Fst) between the three ecotypes for each BnaTCS gene was calculated to estimate the genetic differentiation between populations. We noticed that about 60% of BnaTCS genes were weakly differentiated with Fst less than 0.1 between either two subpopulations. By contrast, there were 1, 5 and 4 genes substantially genetically differentiated with Fst larger than 0.5 in sw_sp (semi-winter vs. spring), sw_wi (semi-winter vs. winter), and wi_sp (winter vs. spring), respectively, in which the largest Fst values were 0.68 (*BnaPRR7d*), 0.70 (*BnaRR4c*), and 0.58 (*BnaRR18b*). Fst values were significantly larger in wi_sp than in sw_sp for BnaHKs and significantly larger in sw_wi than in sw_sp for BnaRRs (Figure 4D).

**Figure 5:**
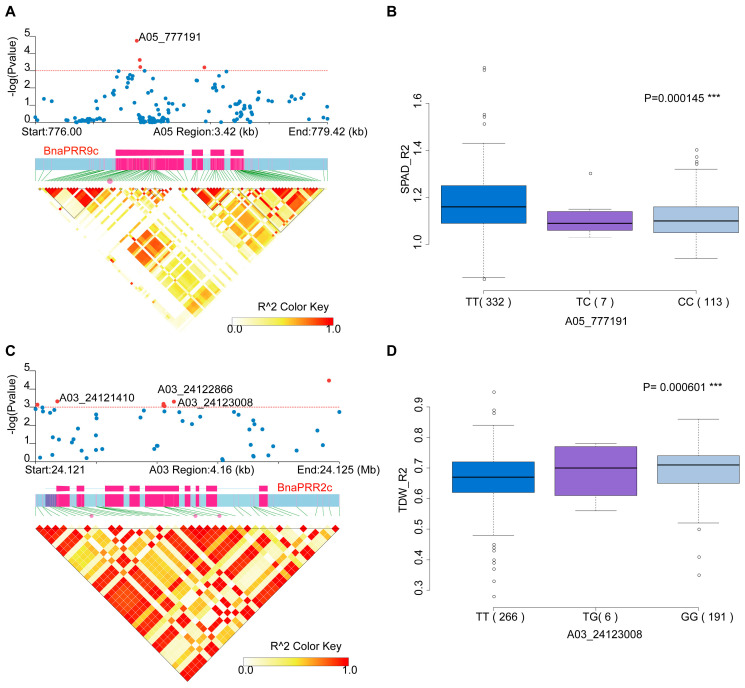
Association mapping analysis of BnaTCS genes in *B. napus* germplasm with 505 collections. (**A**,**C**) Significant association of *BnaPRR9c* with SPAD_R2, and *BnaPRR2c* with TDW_R2 (total dry weight). The upper and lower subgraphs are the local Manhattan plot and the linkage disequilibrium heat map. The SNPs displayed in red are significant association ones, and SNPs labeled are nonsynonymous SNPs located in the exonic region. The exon structure of genes are displayed in rose red. (**B**,**D**) Box plots for SPAD_R2 and TDW_R2 based on the haplotypes of variants in the gene region of *BnaPRR9c* and *BnaPRR2c*.

Furthermore, we investigated the genetic variants of BnaTCS genes in another natural population with 505 *B. napus* accessions (including semi-winter ecotypes and spring ecotypes) [50] and conducted association mapping analysis to explore the genetic effects of BnaTCS genes on salt tolerance. A total of 47 SNPs (MAF > 0.05) and small indels in 24 BnaTCS genes were significantly associated (*p* < 0.001) with the salt-tolerance-related coefficient (STC), which was represented by the suffix “trait_R1” or “trait_R2” and calculated as the ratio of salinity stress response trait value under salt stress conditions to that under normal conditions (Table S10). Among them, 17 SNPs in 12 genes were nonsynonymous. *BnaPRR5e*-*f*, *BnaPRR7c*, and *BnaPRR9a*-*c*, which were homologs of reported salinity negative regulators—*APRR5*, *APRR7*, and *APRR9* [35]—were significantly associated with FYL_R1/R2 (germination rate), FYS_R2 (germination potential), LA_R2 (leaf area), SPAD_R2 (chlorophyll content). *BnaCKI1c*, *BnaCKI2e*, *BnaHP2d*, *BnaPHYAc*, *BnaPRR2c*, *BnaRR18a*, and *BnaRR9a,c* also contained nonsynonymous and significantly associated SNPs. Another two genes, *BnaHK2a* and *BnaHP3b*, whose MAF was about 0.04, were significantly associated with SPAD_R2 and PH_R2 (Plant height), respectively. According to the most significantly associated SNPs of these genes, the trait between the two genotypes was statistically significant according to the ANOVA test (Figure 5).

### 2.5. Cis-Elements Analysis of the BnaTCS Genes

For a better understanding of potential transcriptional regulatory motifs of *B. napus* TCS genes, we identified cis-regulatory elements in the putative promoter regions (2000 bp regions upstream to initiation codon). A number of hormone-related (e.g., ABA, auxin, ethylene, GA, MeJA, and SA) and abiotic stress-related (e.g., anoxic, light, extreme temperature, and wound) elements were discovered, and the most numerous cis-elements were displayed in Figures S7–S9. Among them, the anoxic, ABA, MeJA, and GA-responsive elements were found with high frequency in BnaTCS genes. Notably, light-responsive elements (LREs) were present with multi-copies in most BnaTCS genes. In addition, the auxin-responsive elements (Aux), and drought-inducibility elements (MBS) were found in 72 and 73 BnaTCS genes, respectively, in which auxin-responsive elements (Aux) can bind to auxin response factor (ARF) proteins and confer auxin response. The existence of these cis-elements suggests that the TCS genes may have potential functions in regulating plant development and responding to abiotic stresses.

### 2.6. Expression Analysis of BnaTCS Genes

For a better understanding of the potential function of BnaTCS genes during development and under environmental stimuli, we investigated the dynamic gene expression of BnaTCS genes in various tissues and examined the expression changes under different stress conditions.

#### 2.6.1. Expression Profiles in Various *B. napus* Organs

**Figure 6:**
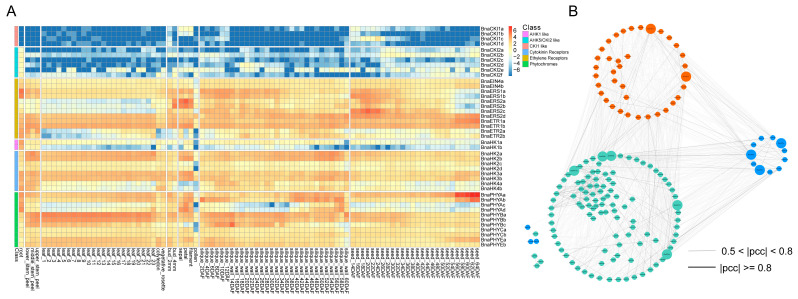
(**A**) Heat map representation for expression of HK(L) genes in various tissues at different development stages of *Brassica napus*. The expression levels of genes are presented using fold-change values transformed to Log_2_ format. The Log2 (fold-change values) and the color scale are shown at the top right of the heat map. Classifications of genes are marked by corresponding colors that are shown in the color legend at the top right. (**B**) The co-regulatory network of BnaTCS genes. Nodes in Orange, blue, and green represent genes of three subfamilies: BnaHK, BnaHP, and BnaRR. The bigger nodes are the ones with the most gene pairs, and the nodes inside the circle are genes that have no strong correlation with other subfamilies. The distinct correlation levels of gene pairs are marked by edge lines with different colors shown at the bottom.

The publicly available expression datasets of 182 BnaTCS genes in 91 tissue samples (including bud, flower, leaf, root, seed, silique, and stem) of rapeseed cultivar ZS11 during seven developmental stages were obtained from the BnIR database [51]. BnaTCS genes are specifically expressed in different tissues of the plant depending on their function. Among them, the transcripts of 15 genes were barely detected, while 18 genes were expressed in all tissues (TPM ≥ 1) (Figure 6A). Almost all cytokinin receptors (*BnaHK2*s, *BnaHK3*s, and *BnaHK4*s), ethylene receptors, and photoreceptors were expressed in all tissues, which implied the potential functions of these BnaHKs in shoot growth, leaf senescence, seed size, germination, root development, and cytokinin metabolism as reported in *Arabidopsis* [22]. Similarly, most BnaHPs and type-B RRs were also globally expressed (Figure S10), which is consistent with previous studies that HPs and type-B RRs act as positive regulators in cytokinin signaling and function in multiple aspects of plant development [25,31]. In addition, part of the TCS genes was organ-specific or had preferential expression. For example, *BnaHK1*s, *BnaHK5*s, *BnaERS1*s, *BnaHP1*s, and *BnaHP5*s showed relatively higher expression levels in roots, which were generally considered the primary organs involved in various abiotic stress responses. *BnaCKI1*s were mainly expressed in flowers and seeds, which is in line with the finding that *CKI1* was mainly transcribed in flowers and was involved in female gametophyte development in *Arabidopsis* [52]. *BnaHP3*s were abundantly expressed in leaves, *BnaPHP1*s were predominantly expressed in roots and seeds, and *BnaRR22*s were only expressed in siliques and seeds.

The majority of TCS genes that were classified into the same subfamilies displayed similar spatio-temporal expression patterns, especially in paralogous gene pairs (Figure 6A and Figure S10). Nevertheless, a small number of subfamilies showed different expression patterns. For instance, in the HP subfamily, *BnaHP4a* was expressed in all detected tissues, but its paralogous gene *BnaHP4b* was barely expressed in flowers and seeds, and in subfamily Type-B PRR, *BnaPRR6a* was expressed in seeds, but the transcription of its paralogous gene *BnaPRR6b* was barely detected in seeds.

Combined RNA-seq datasets were collected from different tissues and different stresses described in the following chapters; we calculated the Pearson correlation coefficients (PCCs) among BnaTCS genes and constructed a co-regulatory network (Figure 6B). There were 1232 gene pairs (|PCC| ≥ 0.5) within 143 BnaTCS genes, of which 53.7% were within three subfamilies and 46.3% were between subfamilies. *BnaHK1*s, *BnaHK4*s, BnaHPs (except *BnaHP4*s), *BnaRR8*s, and *BnaRR12*s had more gene pairs, suggesting their core role and strong interaction in the network. On the contrary, *BnaERS2*s, *BnaHP4*s, *BnaPRR6*s, *BnaRR2*s, *BnaRR5*s, and *BnaTOC1*s were associated with very few genes.

#### 2.6.2. Expression of BnaTCS Genes in Response to Abiotic Stresses

**Figure 7:**
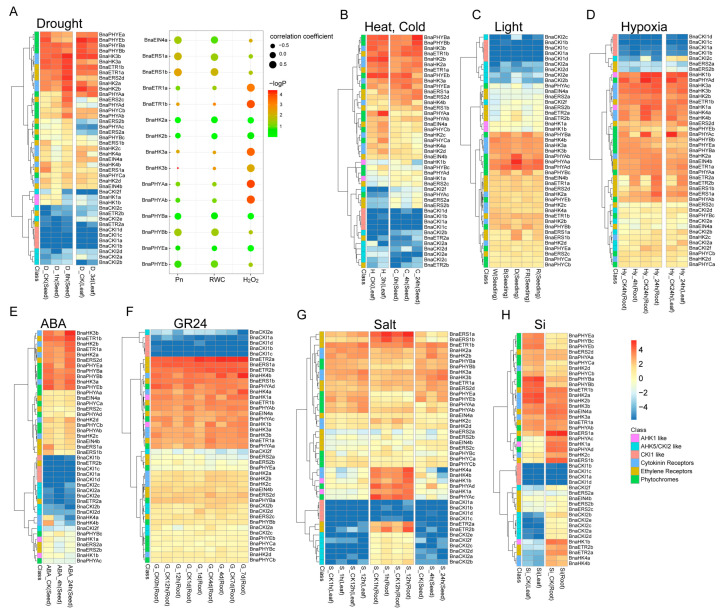
Heat map representation for expression of BnaHK(L) genes under drought (**A**), heat and cold (**B**), light (**C**), hypoxia (**D**), ABA (**E**), GR24 (**F**), salt (**G**), and si (**H**) stress conditions. The bubble diagram at the rightside of (**A**) is the correlation between genes and drought-related traits. The expression levels of genes are presented using fold-change values transformed to Log_2_ format. The Log_2_ (fold-change values) and the color scale are shown at the bottom right of the heat map. Classifications of genes are marked by corresponding colors that are shown in the color legend at the bottom right.

The expression profiles of the BnaTCS genes under different stress conditions including drought, heat, cold, salt, hypoxia, light, Si, ABA, and strigolactones (SLs) induction were utilized to analyze the expression patterns of BnaTCS genes under abiotic stress (Figure 7, Figures S11 and S12).

For drought treatment, the response patterns were complicated. Genes in the same subfamily might be regulated differently, especially in the HK subfamily. As shown in Figure 7, expression levels of *BnaHK2a-b, BnaHK3*s, *BnaETR1*s, *BnaETR2*s, and *BnaPHYA*s were up-regulated at 8 h in seeds, while the remaining cytokinin receptors and ethylene receptors, as well as *BnaPHYCa* were slightly declined at 1 h followed by an increase at 8 h. Only *BnaPHYE*s and *BnaHK1*s were suppressed. Similar patterns were observed in the HP subfamily, in which *BnaHP2b* and *BnaHP3b* were the most significantly up- and down-regulated genes (Figure S11A). Almost all the PRR genes were induced by drought, especially *TOC1* and *PRR5* homologs. A subset of type-A RRs, *BnaRR15*s, *BnaRR5*s, and *BnaRR7*s, were significantly down-regulated (Figure S12A), which was consistent with rice [53], but in contrast with *Arabidopsis* [54]. Homologs of *RR4* in both rapeseed and *Arabidopsis* were marginally down-regulated. This result suggested varied regulative patterns in terms of the species. Furthermore, we checked the expression pattern of TCS genes in another time series transcriptome study [55], in which a co-expression network was constructed based on leaf expression profiles of BnCK (a wild-type rapeseed ‘ZS6’), *BnSGI*-CRISPR and *BnSGI*-overexpression lines under prolonged drought (14 days) followed by re-watering. *SGI* (*Stress and Growth Interconnector*) is a dicotyledon-specific gene and was verified as a positive regulator of drought. In total, 22 out of 66 BnaTCS genes (FPKM ≥ 5 in at least one sample and involved in co-expression analysis) were clustered in modules ‘a’ and ‘b’, which shows a positive correlation with photosynthetic carbon assimilation (Pn) and the relative water content (RWC), and another 22 members were clustered in module ‘y’, which shows a positive correlation with H_2_O_2_ (Figure 7A and Figure S13, Table S11). *BnaHP2c* and *BnaRR10a* were the most relative genes with Pn and RWC, suggesting their potential positive regulation of drought consistently with *SGI*. On the contrary, *BnaPRR5*s, *BnaPRR9*s, *BnaPHYAa*, *BnaETR1*s, *BnaHK3a*, and *BnaRR2*s had a significantly positive correlation with H_2_O_2_, suggesting their potential negative regulation of drought in the regulatory pathway of *SGI*.

Most type-A RRs were significantly suppressed at 24 h by cold treatment (Figure S12B), but several of them were initially transiently induced at 4 h. Expression patterns of *BnaPRR5*s and *BnaPRR9*s were similar to type-A RRs, but their expression returned to a level close to the initial state, whereas type-A RRs decreased to a lower expression level. BnaHK(L)s and BnaHPs were not significantly regulated by cold treatment except for two PHYB and two HP4 homologs. For the high-temperature treatment, a subset of type-A RRs, *BnaRR5-7*s were quickly and slightly suppressed, showing their opposite response patterns with cold. However, under high-temperature stress, expression patterns of most PRR members except *BnaPRR3* and *BnaPRR7*s were consistent with those under cold stress.

Light is a major environmental signal influencing a multitude of steps in plant development, and the phytochromes are photoreceptors that control growth and development in response to environmental cues. Under different light qualities (white, blue, red, and far-red light) [56], the expression levels of *PHYA* homologs were decreased compared to dark conditions. The expression of *PHYB* homologs was enhanced in blue/far-red light but did not change in response to red and white light. Furthermore, compared with the control (dark), *BnaPRR9*s were also up-regulated in response to blue and far-red light and a type-B RR, but *BnaRR14a* was down-regulated.

Hypoxia in the root is one side-effect of waterlogging and may cause multiple problems such as low gas diffusion, changes in redox chemistry, and accumulation of toxic metabolites for plants [57]. After 4 h of hypoxia treatments, transcripts of *BnaERT2*s, *BnaHP4*s, *BnaRR14a, BnaPRR5c-d*, and *BnaRR8*s were up-regulated compared to air-treated controls, while for *BnaHK4s*, *BnaPHYAc*-*d*, *BnaHP1*s, and most type-A RRs, their transcripts were clearly suppressed with down-regulated expressions. The response patterns to the hypoxia treatments in roots and leaves were generally consistent.

Salt stress impacts the growth, development, and production of oilseed rape. Thus, we also examined the effects of salinity on TCS gene expressions. The expression patterns in roots and seeds seemed to be more diverse than that in leaves. In seeds, the expression of *BnaHK2a*-*b, BnaERS2d,* and *BnaPRR7*s was primarily enhanced at 4 h until 24 h. Expression of most type-A RRs was decreased. In roots, in response to salinity treatment, *AHK4* homolog expression was initially suppressed and followed by an increase after 12 h, while *ERS1* and *ETR2* homologs maintained high expression levels. Another chemical substance—Si—is considered a beneficial but non-essential element for plant growth and development [58]. However, TCS genes were not sensitive to Si because no significant change in gene expression was observed between the Si treatment and control.

ABA and SLs are important phytohormones that are involved in the regulation of plant growth, development, and stress responses [59,60]. *AHK4* homologs were continually suppressed by ABA treatment in 24 h. However, in the SL condition (GR24), they kept steady within 24 h and were followed by an increase after 4 days’ treatment. The expression of *BnaHP4a*, *BnaHP4d,* and *BnaRR2a* was up-regulated at 24 h after ABA treatment, while there was no significant change under SL treatment. Almost all Clock PRRs were quickly induced and subsequently repressed 24 h after ABA treatment, but no significant difference was observed in SL conditions. Compared with the control, a subset of type-A RR genes was down-regulated at 24 h in response to both ABA and SL treatments. This is consistent with the participation of type-A RRs in the response of *Arabidopsis* to ABA stress [34]. In addition, the expression of these genes showed an increase after 4 days under SL conditions.

#### 2.6.3. Expression of BnaTCS Genes in Response to Biotic Stresses

**Figure 8:**
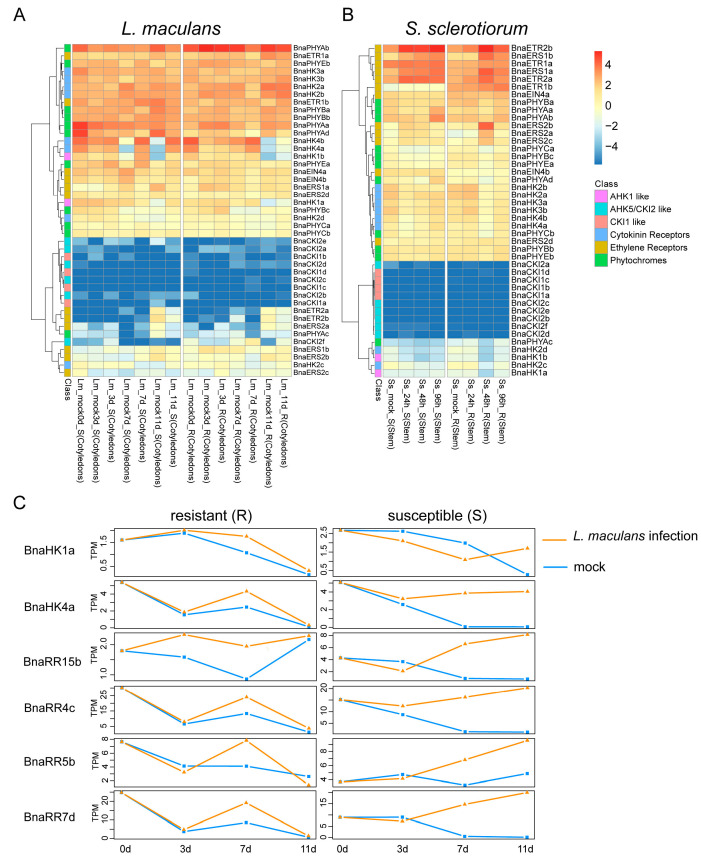
Heat map representation for expression of BnaHK(L) genes under *L. maculans* (**A**) and *S. sclerotiorum* (**B**) infection. The letter “R” and “S” in sample name stand for “resistant” and “susceptible”, respectively. (**C**) Expression patterns of the representative genes that are specific for *L. maculans* response in resistant (R) and susceptible (S).

The hemibiotrophic fungus *Leptosphaeria maculans* and necrotrophic fungus *Sclerotinia sclerotiorum* are the pathogenic microorganisms of blackleg disease and Sclerotinia stem rot, respectively, which cause significant loss of yield in *B. napus* worldwide [61,62]. In response to *L. maculans,* most of the BnaTCS genes exhibited comparatively similar expression levels and patterns between resistant (R) and susceptible (S) cotyledons, as well as between mock-inoculated and *L. maculans* infection, except for the homologs of *AHK1*, *AHK4*, and type-A BnaRRs (especially *BnaRR4*s, *BnaRR5s*, *BnaRR7*s, and *BnaRR15*s) (Figure 8A). The expression trend for one copy of these genes is shown in Figure 8C. As shown, only a few genes (*BnaRR15*s and *BnaRR5c*) were differentially expressed at 3 days post-inoculation (dpi) in both the R and S lines which was consistent with lesion size [61], but the transcription levels of most of these genes were increased to several folds higher than the mock at 7 dpi. Remarkably, in susceptible hosts, expression levels of these genes were still apparently more up-regulated than mock-sprayed samples at 11 dpi, while no significant difference was observed in resistant hosts and their expression was down-regulated at 7 dpi. This indicated that the expression pattern of these genes differed in susceptible and resistant hosts after several days of infection, and they might have functioned in defense against *L. maculans.* Conversely, under *S. sclerotiorum* infection, a difference was observed in ethylene receptors such as *BnaERS1*s, *BnaERS2*s, and *BnaETR2*s. Expression of these genes was up-regulated at 48 h and followed by a great decline in the resistant host. However, in susceptible hosts, the transcripts of these genes were induced at an early stage (24 h), and high expression levels were sustained (Figure 8B), suggesting that these genes may play a vital role in defense against *S. sclerotiorum* in *B. napus.*

## 3. Discussion

In this study, a total of 182 TCS family members were identified in *Brassica napus.* Owing to genome triplication and allopolyploidy of oilseed rape, TCS members in rapeseed were up to an average of 3.5 copies of *Arabidopsis* genes, in which 159 of the 182 members were found to be segmental duplications, whereas only two tandem duplicated gene pairs were found. Therefore, the expansion of TCS genes in rapeseed was mainly attributed to segmental duplications. Furthermore, almost all orthologs of *Arabidopsis* TCS gene were found in rapeseed, except PHYA, ARR13, and APRR8, which might be redundant during rapeseed genome evolution. Compared with some studies on representative plant species such as *Oryza sativa* and *Glycina max*, rapeseed has the highest number of TCS genes. Interestingly, the number of different types of TCS genes varies in fold change among different species. For example, the number of HK and HP genes identified in *B. napus* was about 1.2 times that of *G. max*, but there was more than twice as much of each type of RR gene. Genetic diversity showed significant differences between the three subfamilies, and some members showed substantial genetic differentiation among the three ecotypes, suggesting that their adaptability to the environment was different.

As one of the most important mechanisms providing resistance against biotic and abiotic stresses, expression patterns of the TCS pathway are useful in learning their involvement in coping with environmental stresses. In this study, the expression levels of the BnaTCS gene were investigated in different tissues under normal growth conditions and various stress conditions such as drought, heat, cold, salt, hypoxia, light, Si, ABA, SLs induction, *Sclerotinia sclerotiorum* and *Leptosphaeria maculans* infection. We found the majority of BnaTCS genes had higher expression levels in roots in normal growth (TPM > 1) (Figure 6A and Figure S10). This may be correlated with the fact that cytokinins are synthesized mainly in roots, which are generally regarded as the main organs involved in drought and salt stress. Cytokinin receptors, most HP, and type-B RRs were globally expressed, which was correlated with their potential roles in diverse cytokinin-regulated developmental processes including leaf senescence, seed development, and shoot and root growth [22,23,24,25]. Notably, compared with *Arabidopsis*, there were several genes showed different tissue-specific expression patterns. For example, *AtRR2* had relatively higher expression levels in mature pollen [11], but homologs in rapeseed were the opposite, with lower expression in pollen (Figure S10B). In roots, *BnaCKI2s* were all transcribed (Figure 6A), but ACKI2/AtHK5 was not expressed [11].

Based on the expression pattern analysis of BnaTCSs under multiple abiotic conditions, we found that the regulation of BnaTCS genes showed significant differences in response to different stress factors. During drought, salinity, cold, and heat treatment, most expressed genes were induced or suppressed, while few were sensitive to light, Si, GR24, and hypoxia treatment. BnaRRs were most active in response to stress. Almost all type-A BnaRRs responded to the above stress conditions but differed in response speeds and intensities. Interestingly, the response patterns of a part of HPs and type-A RRs in rapeseed were generally opposite to heat and cold treatments, which were rapidly downregulated by heat but induced by cold. This opposite reaction pattern was also observed in *I. batatas*, but the genes were not identical [18]. In *I. batatas*, a type-B RR *IbRR28* was observably induced upon heat treatment and was suppressed in cold treatment conditions, but no obvious difference was observed for type-B BnaRRs under these two stresses. Of course, there were also several rapeseed PRRs (*BnaTOC1s*, *BnaPRR5s*, and *BnaPRR9s*) that responded uniformly to heat and cold. In addition, BnaTCS expression analysis shows that the response patterns of abiotic stress may be similar or different in different tissues. For example, the responses of BnaTCSs to salt stress were different in leaves, roots, and seeds. *BnaHK4*s were significantly downregulated in seeds but were induced in roots under salinity stress. However, expression patterns of BnaTCSs in leaves and roots were highly consistent under hypoxia conditions.

Drought and high salinity are two important stress factors in rapeseed. It is of great significance to find and study the responsive genes to these two stresses in rapeseed breeding. Interestingly, most of the type-A RRs were downregulated, while most of the PRRs were upregulated in response to both drought and salt stress in rapeseed. Different patterns were observed in *BnaHP4*s, which were downregulated in response to drought but upregulated in response to salt. Meanwhile, similar expression changes were also observed in ‘D_3d (Seed)’ and ’S_24h (Seed)’. Therefore, we speculated that most BnaTCS genes might play similar roles in response to drought and salinity. The expression levels of type-B RRs in rapeseed were relatively stable under different conditions. Among them, *BnaRR1s* and *BnaRR2a*-*b* were up-regulated by drought within 8 h, while *ARR10* and *ARR12* homologs were not regulated, suggesting a different drought signaling regulating pathway from that of *Arabidopsis*. By comparing the expression changes of two samples ’D_8h (Seed)’ and ‘S_4h (Seed)’, which were treated with similar duration, we found the expression levels of *BnaHK2*s and *BnaHK3*s were induced within 10 h by both drought and salt stresses. Conversely, *BnaHK4*s were depressed, which was inconsistent with the *A. thaliana* counterpart. In *Arabidopsis*, these three cytokinin receptors were revealed to function as negative regulators in osmotic stress responses [26]. The different response patterns of *BnaHK4*s with the homologs suggest that there might be functional divergence in their stress responses.

For the drought treatment, the correlation coefficient with drought-tolerance-related traits of BnaTCSs was extracted from a time series co-expression network in our previous study [55]. *BnaRR2a*, *BnaETR1b*, *BnaHK3a*-*b*, *BnaPRR9a*, *BnaPRR5b*, and *BnaTOC1a* were significantly negatively related to RWC, suggesting the regulation roles of drought in the *SGI* (Stress and Growth Interconnector, a positive drought regulator) regulatory pathway. It was amazing that *BnaHP2c* and *BnaRR10a* were remarkably positively related to RWC (cor > 0.8, *p* < 0.01), whereas their *A. thaliana* counterparts both negatively and redundantly control plant responses to drought [34]. Detailed functional characterization is required to define the exact role of these two TCS members. For salt stress, we carried out association mapping analysis based on population SNPs/small Indels and detected significant correlation SNPs with salt tolerance related coefficient in several TCS members, including homologs of salinity negative regulator *BnaHK2a,d, BnaHP3b, BnaPRR5e*-*f*, *BnaPRR7c*, and *BnaPRR9a*-*c.* These results could provide effective information for salt-tolerance resource breeding

Similar to abiotic stress, BnaTCS genes also showed different response patterns to biotic stress stimuli. The homologs of six genes (*AHK1*, *AHK4*, *BnaRR4*s, *BnaRR5*s, *BnaRR7*s, and *BnaRR15*s) were significantly different between R and S lines (Figure 8C), suggesting that they may have potential defensive functions against *L. maculans*. *CKI2* in *Arabidopsis* has been reported to be a positive regulator of bacterial (*Pseudomonas syringae pv* DC3000) and fungal (necrotroph *Botrytis cinerea*) infections [29]. In this study, only *BnaCKI2f* was expressed in cotyledon, and it was up-regulated after 7 days’ *L. maculans* infection compared with the mock-sprayed sample and the resistant sample, while it was not detected at all in the susceptible sample. Conversely, the response difference was observed mainly in ethylene receptors under *S. sclerotiorum* infection.

In this study, we have shown that BnaTCS genes are closely related to the sequences of *Arabidopsis* TCS members (Figure 2), and generally, the expression sites and functions of a great deal of genes are consistent with those of *Arabidopsis*, which is reflected in many studies [63,64]. Thus similar expression patterns of several homologous genes may indicate potentially similar functions. However, the expansion of gene numbers and the presence of two subgenomes may make it difficult to translate functional knowledge from *Arabidopsis* to rapeseed, and even the homologous TCS genes show varied response patterns, indicating that both functional redundancy and functional divergence exist in the homologous genes. More efforts are needed to confirm their function. For the rapeseed TCS genes, the expression analysis is an initial and necessary step. The candidate stress response and related genes provide valuable information for further elucidating the functional role of TCS genes in rapeseed under environmental stress.

## 4. Materials and Methods

### 4.1. Identification and Property Analysis of BnaTCS Genes

The *BnaTCS* genes were identified based on homology searching and protein domain detection. Firstly, *A. thaliana* [2], *B. rapa* [15], *Sorghum bicolor* [17], *Glycine max* [13], and *Oryza sativa* L. [12] TCS protein sequences were retrieved and used as seed sequences to search against *B. napus* var. ZS11 protein sequence dataset (http://cbi.hzau.edu.cn/bnapus/ (accessed on 21 January 2022)) [65] using BLASTP v2.7.1+ [40] program with threshold e-value < 1 × 10^−5^. Hit sequences were then searched by hmmsearch from HMMER v3.3.2 (http://www.hmmer.org/ (accessed on 22 January 2022)) [41] on the basis of three hidden Markov model (HMM) files constructed by identical seed sequences above using HMMER components of hmmbuild. For confirmation of BnaTCS proteins, all putative sequences were further deployed to determine the presence of the specific known conserved domain or motifs of the TCS elements, namely the HK, HATPase, REC, CHASE domain for cytokinin binding (CHASE), cyclic GMP adenylyl cyclase FhlA (GAF), ethylene-binding domain (C2H4), and HPt domains, using different domain databases including Pfam (https://www.ebi.ac.uk/interpro/ (accessed on 1 May 2023)) [42], CDD (https://www.ncbi.nlm.nih.gov/Structure/cdd/wrpsb.cgi (accessed on 1 May 2023)) [43] and SMART (http://smart.embl-heidelberg.de/ (accessed on 1 May 2023)) [44]. In this process, sequences that lacked the specific conserved motifs required for TCS protein function were excluded. BraTCS genes were updated and BolTCS genes were identified using the same method. The protein datasets and annotation of *B. rapa* var. Chiifu and *B. oleracea* var. OX were obtained from the Figshare database [66,67].

The ProtParam tool (https://web.expasy.org/protparam/ (accessed on 24 February 2023)) [68] was used to calculate the molecular weight (MW), isoelectric point (pI), and grand average of hydropathy (GRAVY) of each BnaTCS protein. Subsequently, TMHMM server 2.0 (https://services.healthtech.dtu.dk/service.php?TMHMM-2.0 (accessed on 24 February 2023)) [69] and WoLF PSORT (https://wolfpsort.hgc.jp/ (accessed on 15 February 2023)) [70] were used to predict transmembrane domains and subcellar location, respectively.

### 4.2. Phylogenetic Analysis, Genetic Structure, and Conserved Motifs Analysis

To investigate the genetic structure and phylogenetic relationship between members of two-component systems, multiple sequence alignments of the identified TCS proteins of *B. napus*, *B. rapa*, *B. oleracea* and reported sequences of *A. thaliana* were performed using ClustalW2 tool [71], and the neighbor-joining (NJ) trees were constructed using MEGA 7 [72] with specifying 1000 replicates for the ultrafast bootstrap. Gene clusters of histidine kinase proteins, histidine phosphotransfer proteins, and the response regulators were executed separately for alignment and tree conducting. The trees were displayed by the online tool Interactive Tree of Life (iTOL) v3 (https://itol.embl.de (accessed on 3 March 2023)). The diagrammatic representation of a BnaTCS gene structure combined with a phylogenetic tree was performed using an Integrative Toolkit TBtools [73] based on an annotation file in GFF3 format. A web resource SMART (http://smart.embl-heidelberg.de/ (accessed on 1 March 2023)) was used for the identification, annotation, and display of protein domain architectures.

### 4.3. Chromosome Location, Gene Synteny, and Duplicate Analysis

Gene synteny and duplication provide information about the origin and evolutionary relationship between and among TCS genes of different species. They both depend on the detection of homologous gene pairs. Firstly, whole-genome protein sequences from *B. napus*, *B. rapa*, *B. oleracea* and *A. thaliana* were compared against itself and other genomes using BLASTP (E < 1 × 10^−10^). If a gene had more than one transcript in the annotation, only the representative one was used. Secondly, the MCScanX algorithm [74] was utilized to compute collinear gene pairs based on BLASTP matches and whole-genome gene chromosome location extracted from annotation GFF3 files. All collinear gene pairs among the two-component systems gene family members were detected by extracting the collinearity block within the gene family. The different modes of gene duplication were identified using the DupGen_finder pipeline (https://github.com/qiao-xin/DupGen_finder (accessed on 5 June 2023)) [75], taking *Arabidopsis* as an outgroup for the three Brassica species.

### 4.4. Cis-Acting Regulatory Elements Analysis

According to the location information, the upstream 2000 bp genomic DNA sequences from the transcription start site of BnaTCS genes were extracted from the *B. napus* genome sequence. Then, they were submitted to an online PlantCARE website (http://bioinformatics.psb.ugent.be/webtools/plantcare/html/ (accessed on 7 May 2023)) [76] to predict the putative cis-regulatory elements. Plots were generated with the R (v3.3.2) package ggplot2_3.4.0 (https://cran.r-project.org/web/packages/ggplot2/index.html) (accessed on 7 May 2023).

### 4.5. Expression Analyses Based on RNA-seq Data

Expression data of the BnaTCS genes based on RNA-Seq data sequenced from 91 tissues (bud, flower, leaf, root, seed, silique, and stem) of rapeseed cultivar ZS11 during seven developmental stages were obtained from the BnIR database [51] (http://yanglab.hzau.edu.cn/BnIR/expression_zs11 (accessed on 21 July 2023)).

To examine the expression change of the *BnaTCS* genes under different stress conditions (abiotic, biotic, chemical), we obtained a total of ten expression or RNA-seq datasets from the publicly available database, of which the expression data of leaf and root under salt treatment (Bioproject: PRJNA214511), cotyledons under *L. maculans* infection (Bioproject: PRJNA311316) [61], leaf under *S. sclerotiorum* infection (Bioproject: PRJNA274853), and leaf and root under Si treatment (Bioproject: PRJNA507014) [77] were extracted from the BrassicaEDB database (https://brassica.biodb.org/downloads (accessed on 7 May 2023)) [78], expression data of root under GR24 treatment were obtained from published research [79], and RNA-Seq data of leaves under NaCl, ABA, cold and dehydration treatment (Bioproject: CRA001775) [80], leaf and root under hypoxia treat (Bioproject: PRJNA747283) [57], seedlings under different light qualities (white, blue, red, and far-red light) (Bioproject: PRJNA658388) [56], and leaf of rapeseed under drought, heat treat (Gene Expression Omnibus: GSE156029) [81] were downloaded from the SRA-NCBI database. Subsequently, we conducted transcriptome analysis and calculated the expression value based on the RNA-Seq data. Reads were quality checked and mapped to the reference genome using Hisat2 [82]. HTSeq was employed for read counts of each gene [83]. The expression levels in all aforementioned samples were measured by transcripts per million mapped reads (TPM) in this study using a local Perl program.

### 4.6. Nucleotide Diversity

Nucleotide diversity (π) and population fixation statistics (FST) across three genetic clusters for each gene were calculated using VCFtools v0.1.13 (https://vcftools.github.io (accessed on 12 July 2023)). A family-based association mapping analysis was conducted by EMMAX with a mixed linear model [84].

## 5. Conclusions

In summary, we identified 182 TCS genes including 43 HK(L)s, 16 HPs, and 123 RRs in rapeseed. The protein classifications, gene structures, conserved domains, phylogenetic relationships, cis-acting element, gene duplication events, and genetic variations were investigated in detail to provide comprehensive information on the TCS family in rapeseed. We also focused on the response patterns of the TCS genes to various abiotic or biotic stresses and screened out numerous candidate stress-responsive genes in rapeseed. The expression pattern of the rapeseed TCS gene under stresses provides a reference control for other polyploid species. Furthermore, by family-based association mapping analysis, some SNPs/genes were found to be significantly associated with salt-tolerance-related traits. It lays a foundation for future research on gene functions and genetic breeding. This is the first genome-wide study of TCS genes in polyploid plants, which provides ideas and methods for studying stress responses in other plants. The results of this study provide important information for the function and regulation of TCS genes in *B. napus*, which will help to better understand the signal transduction pathways and improve the stress tolerance of this plant.

## Figures and Tables

**Table 1:** Gene pairs within *A. thaliana*, *B. rapa*, *B. oleracea*, and *B. napus*.

Gene Family ^1^	Gene Pairs	Number of Genes Involved in Gene Pairs	Percentages (%)
AHK(L)/BraHK(L)	17	14/17	87.5/81.0
AHK(L)/BolHK(L)	19	14/19	87.5/86.4
BraHK(L)/BnaAHK(L)	29	20/21	95.2/100
BolHK(L)/BnaCHK(L)	34	21/21	95.5/95.5
AHP/BraHP	6	4/6	66.7/75.0
AHP/BolHP	6	4/6	66.7/75.0
BraHP/BnaAHP	15	8/8	100/100
BolHP/BnaCHP	15	8/8	100/100
ARR/BraRR	67	28/54	84.8/91.5
ARR/BolRR	66	28/55	84.8/84.6
BraRR/BnaARR	158	53/54	98.8/93.1
BolRR/BnaCRR	168	62/62	95.4/95.4

^1^ AHK(L), BraHK(L), and BolHK(L) represent HK genes from *Arabidopsis thaliana*, *Brassica rape*, and *Brassica oleracea*, respectively. BnaAHK(L) and BnaCHK(L) represent HK genes from subgenome A and C of *Brassica napus.* The abbreviation for HP and RR genes are similar as described above.

## Data Availability

All relevant data are presented within the paper and its Supplementary Files.

## Supplementary Materials

The supporting information can be downloaded at: https://www.mdpi.com/article/10.3390/ijms242417308/s1. References [85,86,87,88,89,90] are cited in the supplementary materials.
