# Supplementary material for: Two-Component System Genes in Brassica napus: Identification, Analysis, and Expression Patterns in Response to Abiotic and Biotic Stresses
Source: Int J Mol Sci. 2023 Dec 9;24(24):17308. doi: 10.3390/ijms242417308 (PMC10743665; doi:10.3390/ijms242417308)
Supplement: Supplementary file 1 [file ijms-24-17308-s001.zip › ijms-2720240-supplementary files/Supplementary Figures.pdf]

Supplementary Figures:

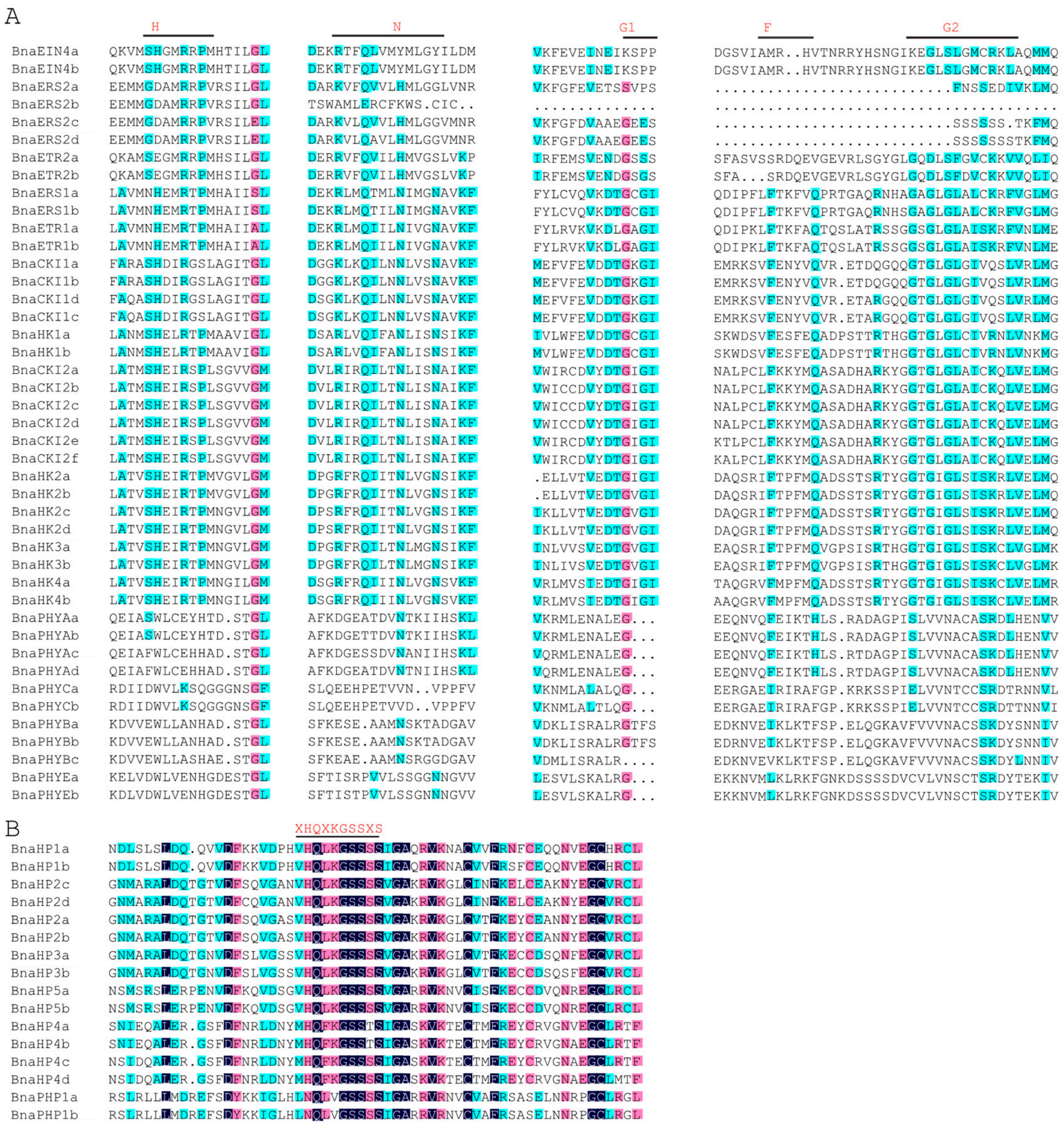

Figure S1. Amino acid sequence alignment of TCSs in rapeseed. (**A**) Histidine kinase (like); (**B**) Hpt . The conserved motifs were marked above the alignments.

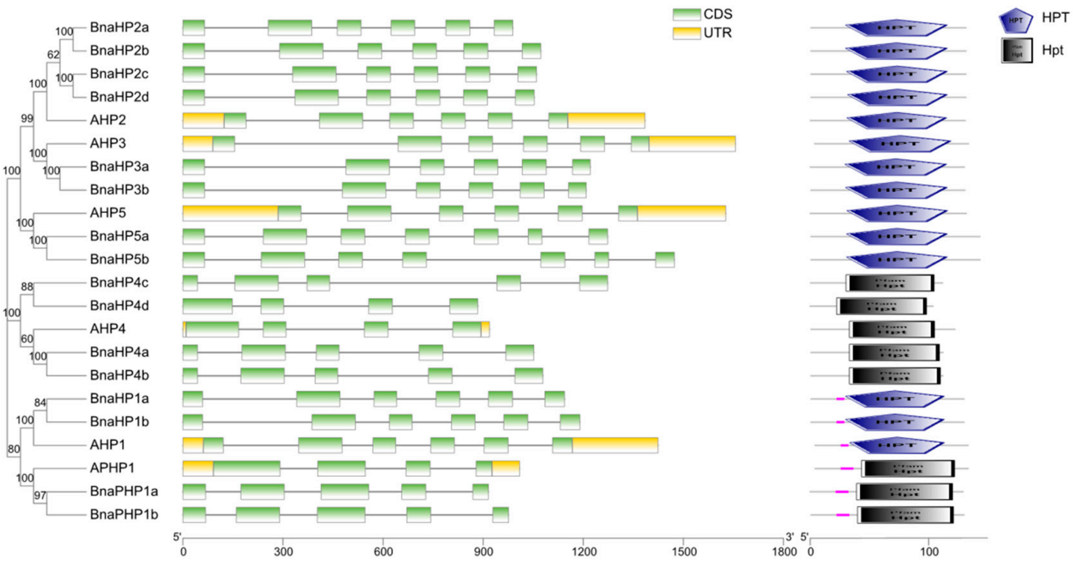

Figure S2. Diagram representations of the gene structures and primary domain structures of BnaHPs.

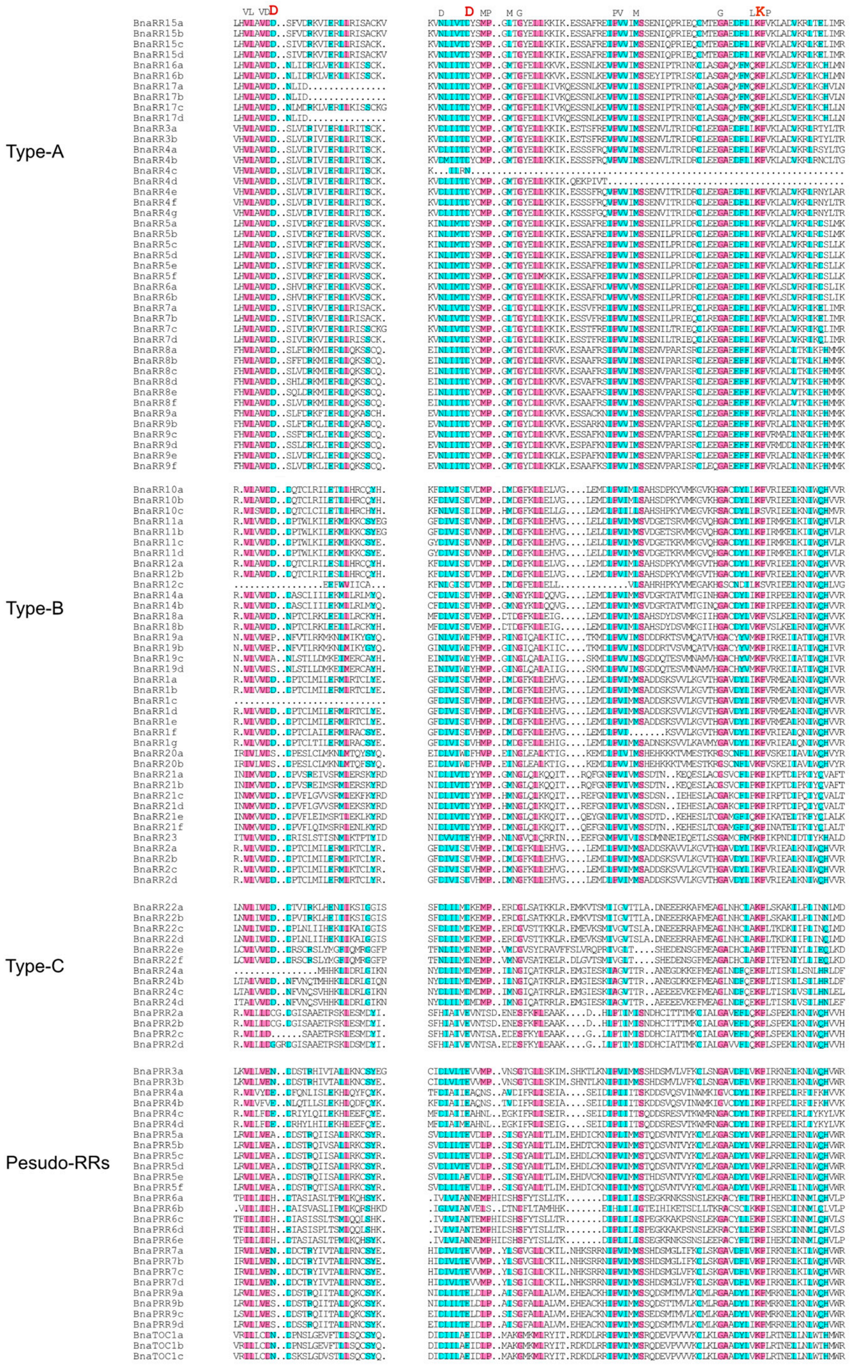

Figure S3. Amino acid sequence alignment of RRs in rapeseed. The conserved motifs was also marked above the alignments.

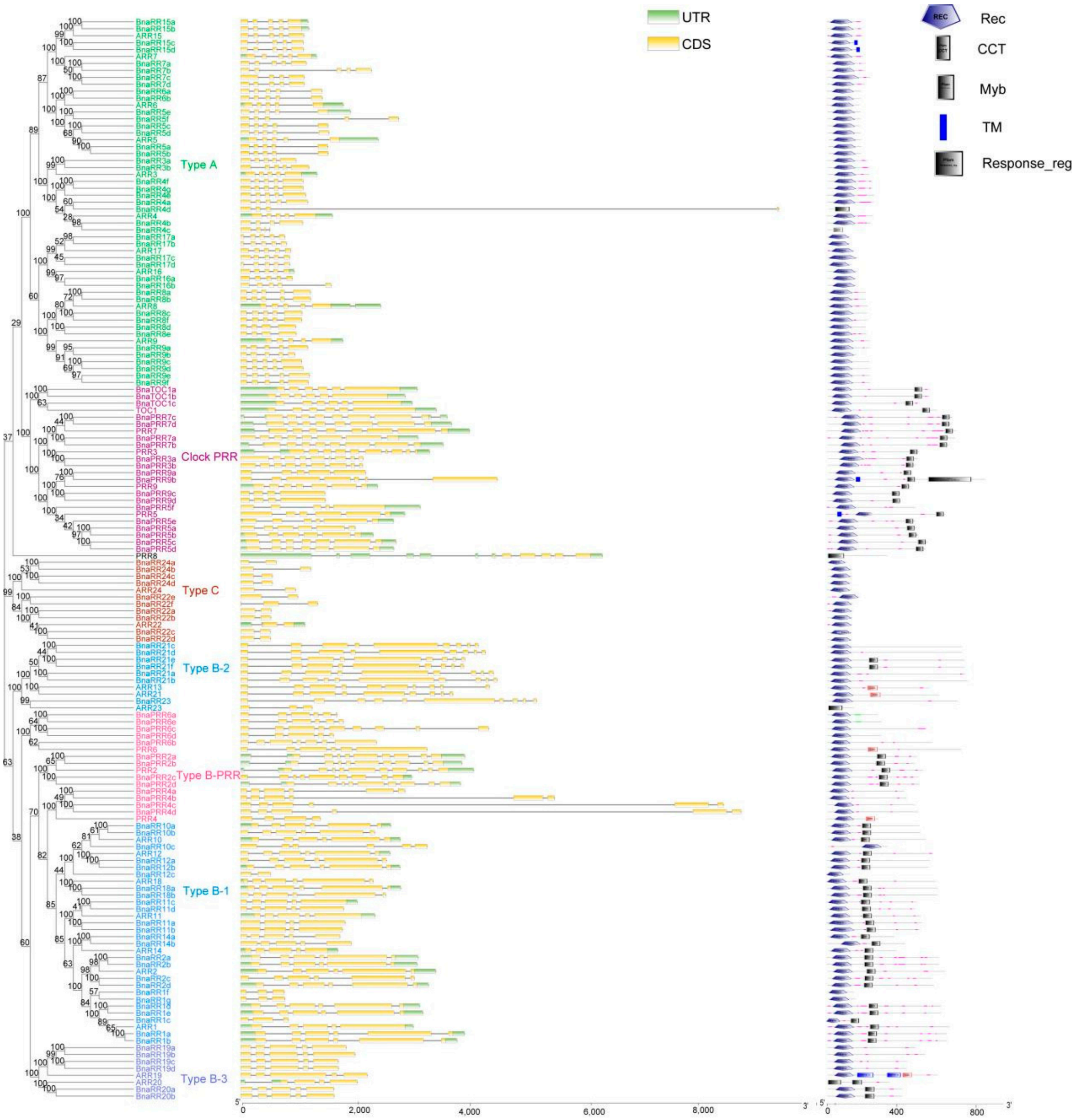

Figure S4. Diagram representations of the gene structures and primary domain structures of RR genes in rapeseed.

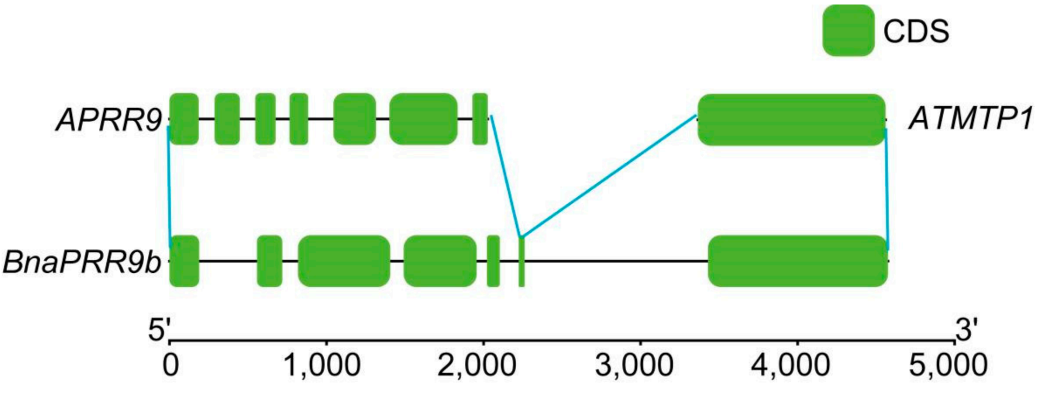

Figure S5. Gene structure of *BnaPRR9b*

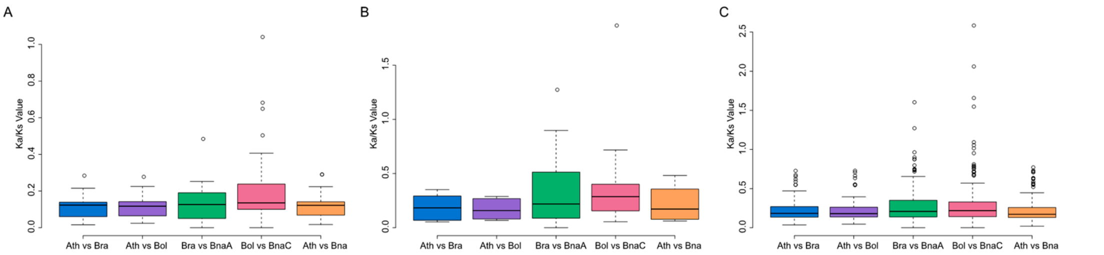

Figure S6. Ka/Ks of HKs (**A**), HPs (**B**), and RRs (**C**) orthologous genes between *A. thaliana*, *B. rapa*, *B. oleracea*, and *B. napus*.

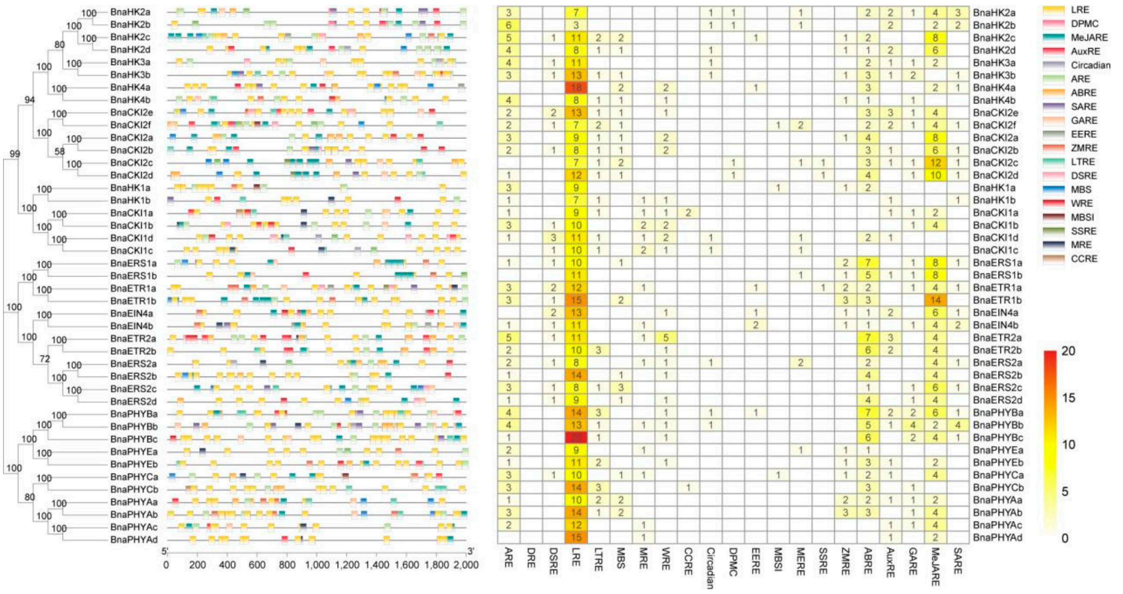

Figure S7. Cis-elements in putative promoter regions of HK(L) genes in rapeseed. Heat map on the right representation for numbers of several hormone-related and abiotic stress-related elements.

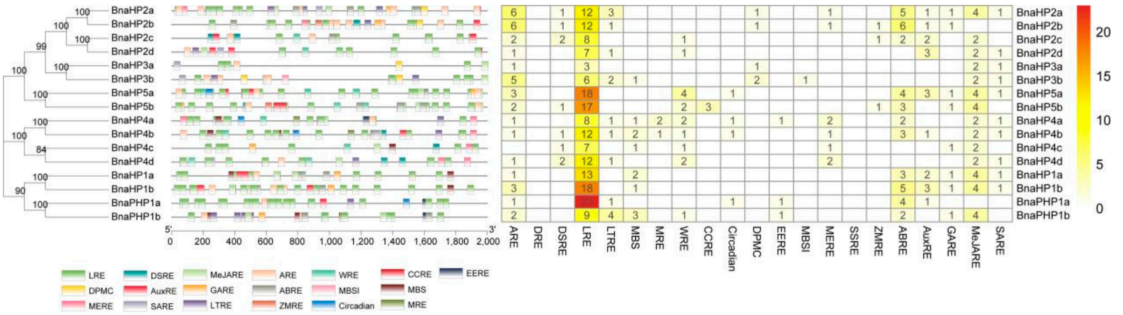

Figure S8. Cis-elements in putative promoter regions of HP genes in rapeseed. Heat map on the right representation for numbers of several hormone-related and abiotic stress-related elements.

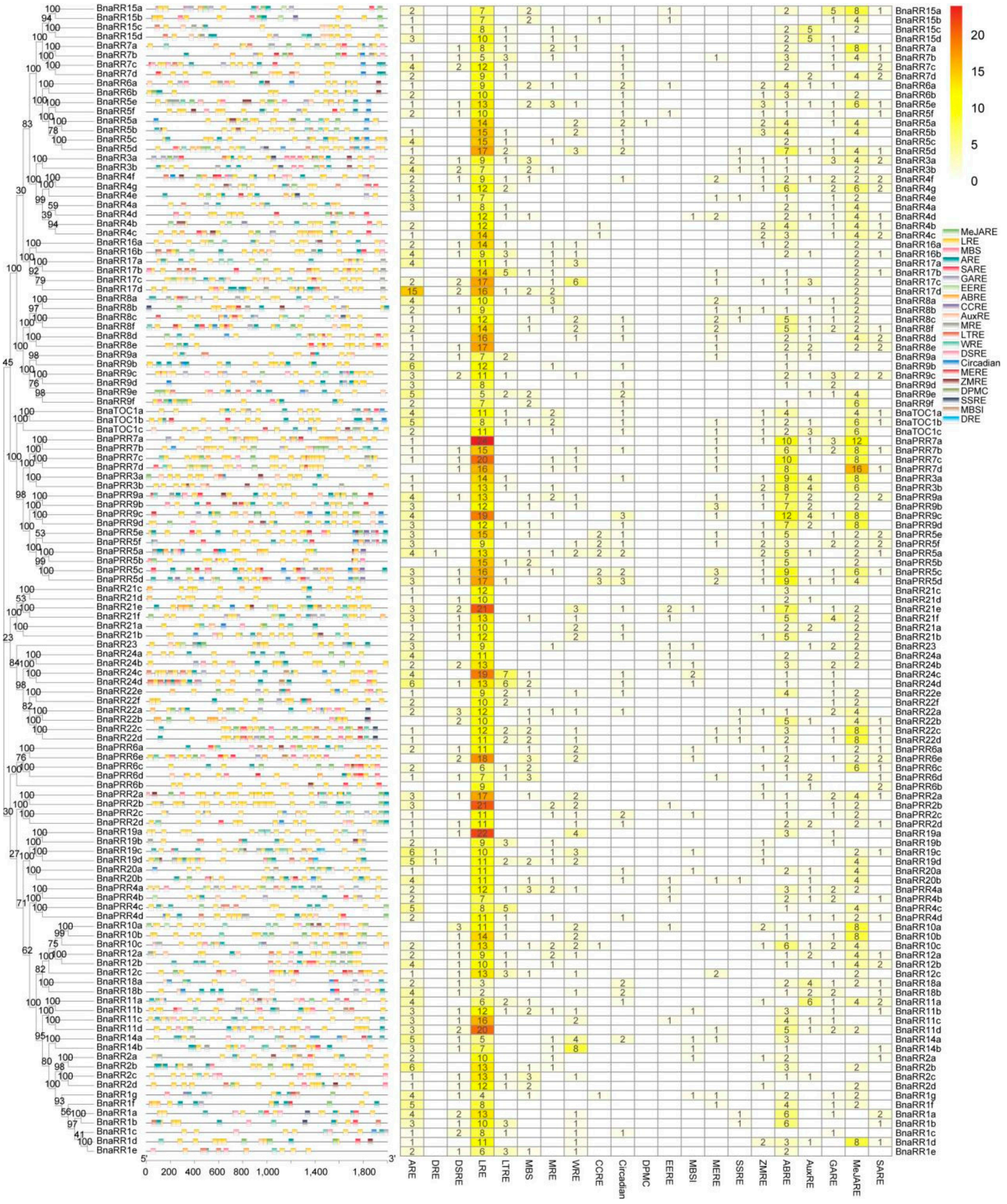

Figure S9. Cis-elements in putative promoter regions of RR genes in rapeseed. Heat map on the right representation for numbers of several hormone-related and abiotic stress-related elements.

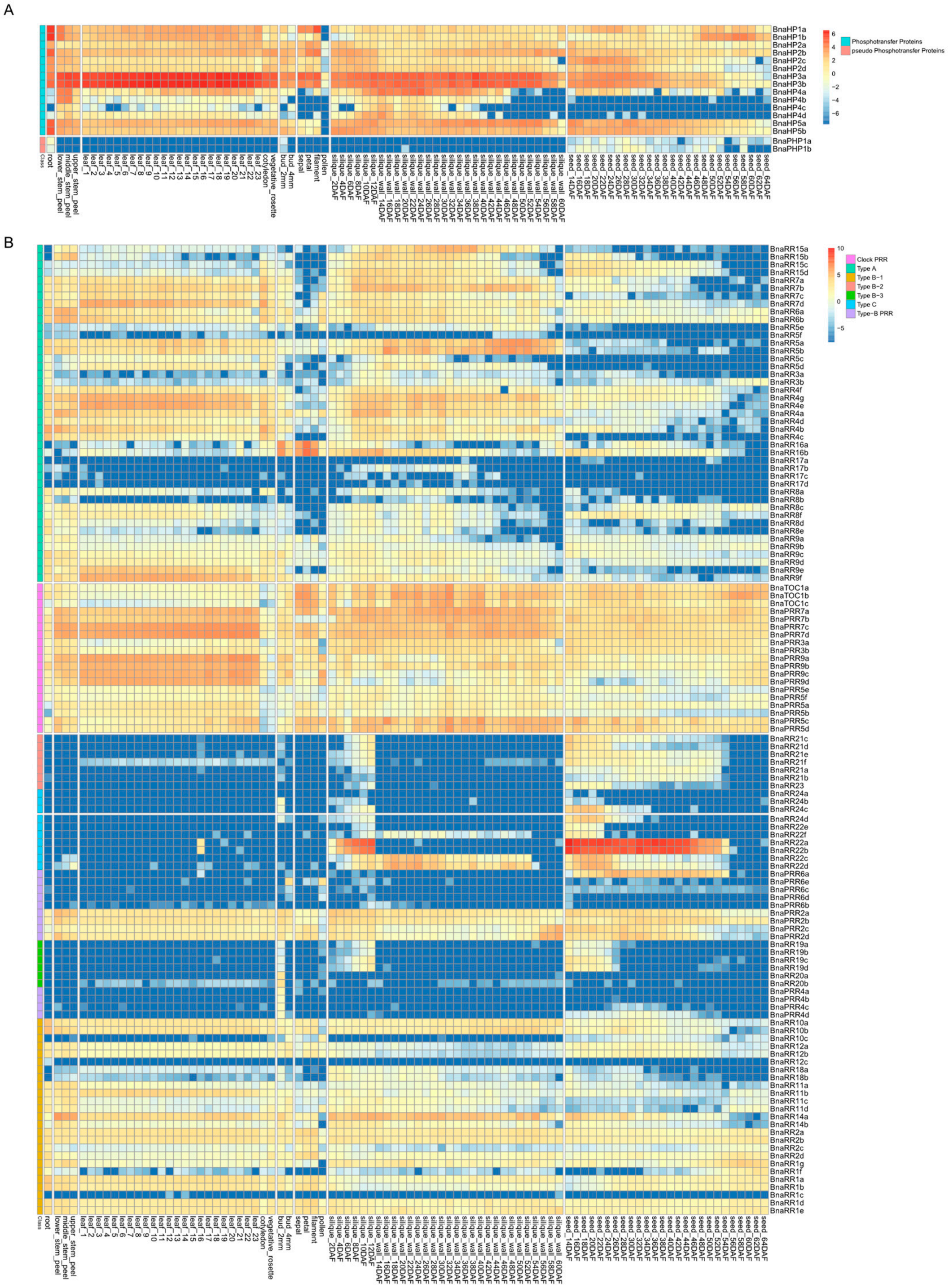

Figure S10. Heat map representation for expression of (**A**) HP, and (**B**) RR genes in various tissues at different development stages of *Brassica napus*.

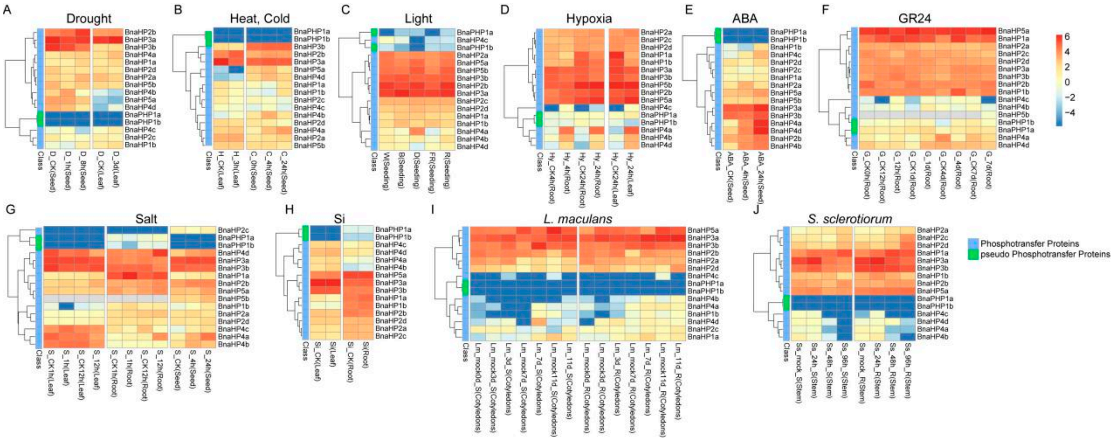

Figure S11. Heat map representation for response patterns of BnaHP genes under drought (**A**), heat and cold (**B**), light (**C**), hypoxia (**D**), ABA (**E**), GR24 (**F**), salt (**G**), si (**H**), *L. maculans* (**I**), and *S. sclerotiorum* (**J**) stress conditions.

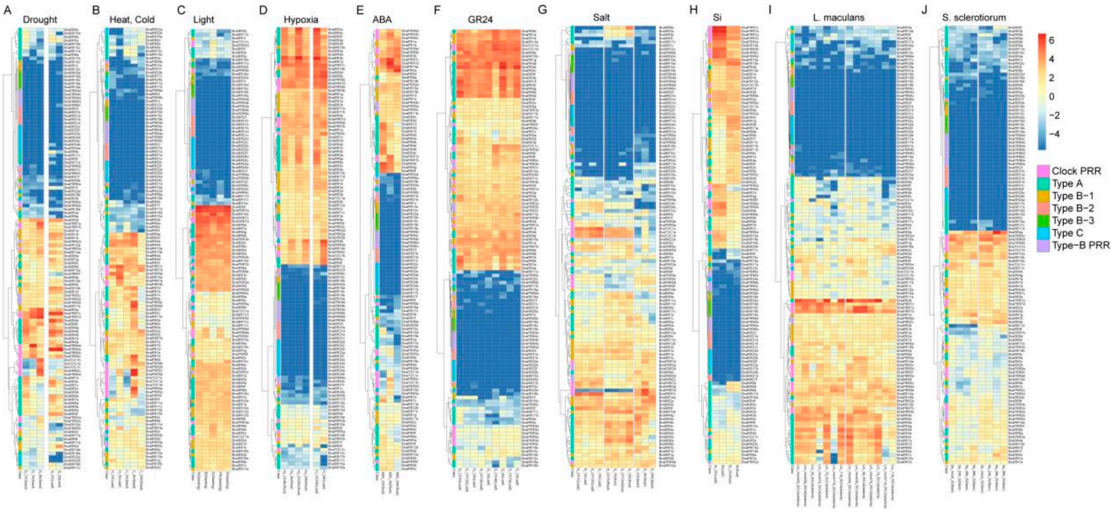

Figure S12. Heat map representation for response patterns of BnaRR genes under drought (**A**), heat and cold (**B**), light (**C**), hypoxia (**D**), ABA (**E**), GR24 (**F**), salt (**G**), si (**H**), *L. maculans* (**I**), and *S. sclerotiorum* (**J**) stress conditions.

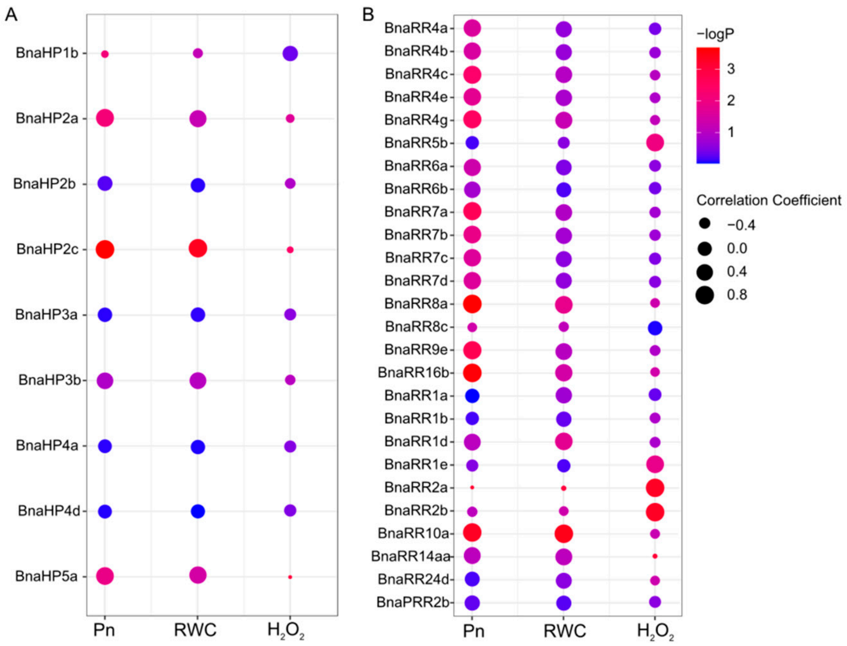

Figure S13. The correlation of (**A**) BnaHPs, and (**B**) BnaRRs with drought related traits.
